# The Global Economic Burden of Tuberculosis: Regional Disparities and Implications

**DOI:** 10.1093/ofid/ofag014

**Published:** 2026-01-29

**Authors:** Ji-Chun Wang, Qin-Yan Zuo, Jin-Xin Zheng, Wen-Wen Lv, Wei Wang, Shun-Xian Zhang

**Affiliations:** Department of Science and Technology, Chinese Center for Disease Control and Prevention, Beijing, China; Department of Science and Technology, Chinese Center for Disease Control and Prevention, Beijing, China; Clinical Research Center, Longhua Hospital, Shanghai University of Traditional Chinese Medicine, Shanghai, China; National Key Laboratory of Intelligent Tracking and Forecasting for Infectious Diseases, NHC Key Laboratory of Parasite and Vector Biology, WHO Collaborating Centre for Tropical Diseases, National Center for International Research on Tropical Diseases, National Institute of Parasitic Diseases of Chinese Center for Disease Control and Prevention, Shanghai, China; Quality Control Center, Clinical Medicine Research Institute, Shanghai Jiao Tong University School of Medicine, Shanghai, China; Editorial Office, National Health Commission Key Laboratory of Parasitic Disease Control and Prevention, Key Laboratory of Jiangsu Province on Parasite and Vector Control Technology, Jiangsu Institute of Parasitic Diseases, Wuxi, Jiangsu, China; Clinical Research Center, Longhua Hospital, Shanghai University of Traditional Chinese Medicine, Shanghai, China

**Keywords:** economic burden, global disparities, public health policy, tuberculosis, value of a statistical life year

## Abstract

**Background:**

The global macroeconomic impact of Tuberculosis (TB) lacks comprehensive quantification using standardized economic frameworks.

**Methods:**

This analysis utilized data from the Global Burden of Disease (GBD) 2021 to assess the economic burden of TB through the lens of the Value of a Statistical Life Year (VSLY) framework, integrating willingness-to-pay-based economic measures. The study monetized the total disability-adjusted life years (DALYs) associated with TB to estimate overall welfare losses. Country-specific gross domestic product (GDP) data, adjusted for purchasing power parity (PPP), were sourced from the World Bank and integrated with DALY estimates to calculate the Value of Lost Welfare (VLW).

**Results:**

In 2021, the global economic cost of TB, expressed as VLW, was estimated at US$1.98 trillion (95% uncertainty interval [UI]: 1.62, 2.45), representing 1.29% (95% UI: 1.05, 1.60) of global GDP. The economic impact of TB was disproportionately distributed across regions, with countries of low Socio-Demographic Index (SDI) facing the most severe burden, where VLW represented 7.83% (95% UI: 5.70, 11.20) of GDP. Lower-middle SDI regions experienced a VLW impact of 5.28% (95% UI: 4.30, 6.58). Sub-Saharan Africa (US$406.2 billion; 8.40% of GDP) and South Asia (US$822.5 billion; 5.70% of GDP) were identified as the most economically affected super-regions. India bore the highest absolute economic burden (US$477.5 billion). In contrast, high-SDI countries demonstrated a VLW-to-GDP ratio of just 0.10% (95% UI: 0.09, 0.12).

**Conclusions:**

Prioritizing TB control in economic policy is urgently needed. Equitable resource allocation to high-burden regions is vital to alleviate the disease's economic consequences and improve global health.

## Graphical Abstract

**Graphical Abstract:**
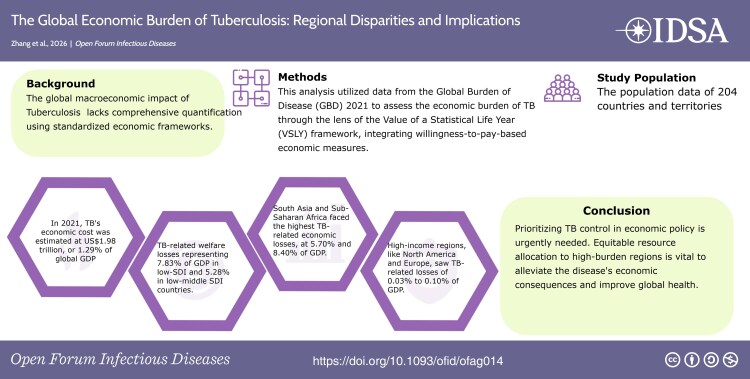
This graphical abstract is also available at Tidbit: https://tidbitapp.io/tidbits/the-global-economic-burden-of-tuberculosis-regional-disparities-and-implications?utm_campaign=tidbitlinkshare&utm_source=ITP

Tuberculosis (TB) is a persistent and debilitating infectious disease primarily caused by *Mycobacterium tuberculosis* (Mtb) strain. It follows a protracted course marked by significant disability and mortality. Its characteristic symptoms—chronic cough, bloody sputum, substantial weight loss, and recurrent fever—not only impair physical health but also reduce quality of life, diminish productivity, and exert substantial pressure on healthcare systems [1–3]. In 2023, global TB incidence reached an alarming 10.8 million new cases, while the disease claimed 1.25 million lives, including 161 000 deaths among people living with Human Immunodeficiency Virus (HIV) [1, 4]. The Corona Virus Disease 2019 (COVID-19) pandemic set back global TB control, causing diagnostic and treatment delays that fueled case resurgence and tragically reestablished TB as the world's leading infectious killer [5].

Although recent advancements in TB treatment, such as the introduction of bedaquiline, delamanid, and pretomanid, have marked significant progress in addressing multidrug-resistant (MDR-TB) and extensively drug-resistant (XDR-TB) forms of the disease, TB continues to impose a substantial global burden, particularly in low- and lower-middle-income regions [6–8]. Persistent TB control challenges—including diagnostic delays, limited healthcare access, and poor treatment adherence—are compounded by comorbidities and socioeconomic inequities, sustaining TB as a major public health crisis and a barrier to global health equity and development goals [1, 4, 9].

At the individual and household level, TB creates a significant financial burden, both in terms of direct medical costs and the broader indirect economic impacts. These include loss of income due to illness, the economic cost of caregiver absenteeism, and the long-term depletion of human capital, particularly in cases of MDR-TB and XDR-TB [10]. The World Health Organization (WHO) has estimated that nearly 50% of households affected by TB are pushed into catastrophic health expenditures, with this financial strain disproportionately affecting individuals in low-income settings [11]. On a larger scale, TB exerts considerable pressure on national public health infrastructures, undermining the capacity and efficiency of health systems. TB disproportionately affects the economically active population, reducing labor supply and hindering economic growth. Its societal impact—including educational disruption, impaired skill development, and social stigma—reinforces poverty cycles and deepens inequality. Consequently, TB not only presents a direct health threat but also creates sustained socioeconomic barriers, particularly in resource-limited settings, underscoring the imperative for integrated control strategies that simultaneously address both health and economic dimensions.

The Global Burden of Disease (GBD) 2021 study offers a standardized methodology, employing disability-adjusted life years (DALYs) to enable cross-national health comparisons and inform policy prioritization. However, its exclusive focus on health-related loss limits its capacity to fully reflect the broader economic repercussions of diseases, thereby restricting its utility in fiscal policy and resource allocation discussions [12–19]. In response to this gap, the Value of a Statistical Life Year (VSLY) framework has been introduced, offering a means to translate DALYs into monetized welfare losses. This approach improves the economic relevance of health assessments by converting health burdens into tangible economic terms. The VSLY method has been successfully applied in global economic burden studies of various conditions, such as stroke, cancer, and preterm birth, significantly enhancing the policy applicability of disease burden data [20–24]. Despite its potential, a comprehensive, systematic economic evaluation of TB that integrates the VSLY approach on a global scale remains absent, limiting the full understanding of the economic impact of TB and hindering its incorporation into global economic and health policies [20–24].

This research drew upon data from the GBD 2021, incorporating essential macroeconomic indicators—such as per capita gross domestic product (GDP), population size, and life expectancy—sourced from the World Bank. These variables were integrated into a VSLY framework to model the economic impact of TB. Specifically, the study quantified the economic welfare losses linked to TB, referred to as the Value of Lost Welfare (VLW), across 204 countries and territories in 2021. By addressing a critical gap in existing literature, it provided the first macroeconomic perspective on TB's economic impact. The findings delivered evidence-based insights to guide targeted interventions, optimize resource allocation, and enhance returns on global TB control investments, advocating for an economically-informed approach to disease management.

## METHODS

### Data

This study integrated data from several standardized global sources to examine the economic burden of TB. Key indicators of TB burden, such as DALYs, life expectancy, and population data disaggregated by age, sex, and region, were sourced from the GBD 2021 study, coordinated by the Institute for Health Metrics and Evaluation (IHME) at the University of Washington. This dataset covered 204 countries and territories and provided a comprehensive view of the global TB impact (https://vizhub.healthdata.org/) [12, 13, 25]. Economic data, including GDP per capita adjusted for purchasing power parity (PPP) in constant 2021 international dollars, were retrieved from the World Bank's World Development Indicators database (https://databank.worldbank.org/databases) [20–25]. To translate health impacts into economic terms, the baseline value of a statistical life (VSL) for 2021 in the United States was obtained from the official estimates provided by the United States Department of Transportation (https://www.transportation.gov) [25, 26]. All datasets were adjusted to align with 2021 metrics, with monetary values standardized to the same year to ensure consistency and accuracy across the various data sources. This robust integration of health and economic data allowed for a more comprehensive analysis of TB's impact, providing essential insights for policy formulation and resource allocation.

## CALCULATION OF TB-RELATED VSL AND ECONOMIC BURDEN FOR 204 COUNTRIES AND TERRITORIES

To accurately assess the future economic burden of deaths and disabilities related to TB, a discount rate was used to calculate the present value of future losses [20, 24, 25, 28, 29]. The specific formula is as follows:


$$\mathrm{VS}L_{a,i,t}={\mathrm{VSL}}_{\;p,\mathrm{UAS}}{\left(\frac{YC_{i}}{YC_{\mathrm{USA}}}\right)}^{\mathrm{IE}\text{-}\mathrm{VSL}}$$

$\mathrm{VS}L_{a,i,t}$ represents the VSL at a specific age (*a*), in a given country (*i*), and during a specific year (*t*).

$\mathrm{VS}L_{\;p,\mathrm{USA}}$ refers to the peak value of the VSL for the United States.

$YC_{i}$ refers to the GDP per capita of country or region (*i*) in 2021, which is equivalent to GDP, PPP (constant 2021 international dollars) for country *i*.

$YC_{\mathrm{USA}}$ represents the GDP per capita of the USA in 2021, it is equivalent to GDP, PPP (PPP) (constant 2021 international $) for country_USA.


$$\mathrm{VSL}Y_{a,i,t}={\mathrm{VSL}}_{a,i,t}\times f(a{)}_{i}\times \frac{-r}{e^{r(a-LE_{a,i,t})-1}}$$

$\mathrm{VSL}Y_{a,i,t}$ represents the economic loss associated with each year of life lost (ie, a component of DALY) due to TB at a specific age (*a*), in a given country or region (*i*), and during a specific year (*t*).

$\mathrm{VL}W_{a,i,t}$ represents the VSLY, indicating the economic welfare loss caused by TB at a specific age group (*a*), in a given country or region (*i*), and during a specific year (*t*).

f(a) is an age-related function that adjusts the VSL based on age. The economic loss due to death at different age groups carries varying weights .

*r* represents the discount rate, which is the factor used to convert future health losses into present value. Typically set at 3.00% [20, 24, 30].

$LE_{a,i,t}$ represents life expectancy, indicating the average remaining years of life for a specific age group (*a*) in a given country (*i*) and time point (*t*).

*a* represents the age of an individual, used in this formula to calculate the years of life lost at a specific age group.


$$\mathrm{VL}W_{a,i,t}={\mathrm{VSLY}}_{a,i,t}(\mathrm{YL}L_{a,i,t}+\int_{a}^{LE_{a,i,t}}e^{-r(x-a)}dx)$$

$\mathrm{VL}W_{a,i,t}$ represents the VSLY, indicating the economic welfare loss caused by TB at a specific age (*a*), in a given country or region (*i*), and during a specific year (*t*).

$\mathrm{VSL}Y_{a,i,t}$ represents the economic loss associated with each year of life lost (ie, a component of DALY) due to TB at a specific age (*a*), in a given country or region (*i*), and during a specific year (*t*).

$\mathrm{YL}L_{a,i,t}$ represents the health years lost due to deaths caused by TB at a specific age (*a*), in a given country or region (*i*), and during a specific year (*t*).

$LE_{a,i,t}$ represents the life expectancy at a specific age (*a*) in a given country (*i*) and time point (*t*), indicating the average remaining years of life for an individual at that age.

*r* represents the discount rate is a factor used to convert future health losses into present value. In line with common practice, this study applies a rate of 3.00% [26].


$$\mathrm{GD}P_{i}(\text{\%})\;=\;(\mathrm{VL}W_{a,i,t}/\mathrm{GD}P_{i})\;\times \;100\text{\%}.$$

$\mathrm{VL}W_{a,i,t}$ represents the economic loss associated with each year of life lost (a component of DALY) due to TB at a specific age (*a*), in a particular country or region (*i*), and during a given year (*t*).

GDP*_i_* refers to the gross domestic product of country iii in a specific year.

### Sensitivity Analyses

Sensitivity analyses using IE values of 0.55 and 1.50 were conducted to assess the impact of income-driven variations in willingness to pay on the outcomes [22, 23]. This approach ensured the robustness and policy relevance of our estimates across different economic contexts.

### Statistical Analysis

For missing GDP (PPP) data from the 204 countries and territories in the study, the following imputation logic methods were applied: First, any missing values for the year 2021 were filled using the median of data from the preceding and following 3–5 years; if no subsequent data were available, the most recent historical data were used. Second, for missing data in non-sovereign regions, values from sovereign countries at the same socio-demographic index (SDI) level were borrowed, or the median of neighboring countries at the same SDI level was applied. In addition, the DALY data were extracted from the GBD 2021 database, accompanied by 95% uncertainty intervals (UIs) [9, 10]. In line with this, the VSLY valuation intervals also incorporated these uncertainty intervals [12, 13]. This ensured that the economic estimates reflected the inherent variability in the data, providing more robust and credible insights for global TB burden assessments and policy formulation [20, 21, 24].

All data processing and computational analyses in this study were conducted using the RStudio Integrated Development Environment (RStudio IDE, RStudio PBC, Boston, Massachusetts, USA). The research design, data handling, result presentation, and other stages of the analysis were rigorously aligned with the Consolidated Health Economic Evaluation Reporting Standards (Supplementary) [31].

## RESULTS

### Global

In 2021, the VLW attributable to TB across 204 countries and regions was estimated at approximately $1.98 trillion (95% UI: 1.62, 2.45), which equated to about 1.29% of global GDP (95% UI: 1.05, 1.60) (Table 1).

**Table 1.** VLW and VLW/GDP by GBD Regions in 2021 for TB, Generated Using IE of the VSL at 1.00

Region	VLW Region (millions) 95% UI	VLW/GDP (%) 95% UI
Global	1 976 035.11 (1 619 024.68, 2453634.82)	1.29 (1.05, 1.60)
High SDI	62 379.74 (52 522.06, 75232.63)	0.10 (0.09, 0.12)
High-middle SDI	233 505.33 (196 838.99, 280787)	0.46 (0.39, 0.55)
Low SDI	148 363.85 (107 937.88, 212262.29)	7.83 (5.70, 11.2)
Low-middle SDI	1 037 182.38 (845 422.11, 1293617.17)	5.28 (4.30, 6.58)
Middle SDI	494 603.81 (416 303.64, 591735.73)	2.38 (2.01, 2.85)
Central Europe, Eastern Europe, and Central Asia	90 700.55 (79 238.96, 103640.42)	0.60 (0.52, 0.68)
High-income	45 144.28 (39 707.31, 50181.16)	0.08 (0.07, 0.09)
Latin America and Caribbean	63 427.01 (54 536.56, 76796.22)	0.54 (0.46, 0.65)
North Africa and Middle East	35 043.23 (25 953.24, 51538.87)	0.38 (0.28, 0.56)
South Asia	822 493.44 (699 705.48, 1002010.12)	5.70 (4.85, 6.95)
Southeast Asia, East Asia, and Oceania	513 012.91 (420 599.94, 630306.55)	1.30 (1.06, 1.60)
Sub-Saharan Africa	406 213.68 (299 283.19, 539161.47)	8.40 (6.19, 11.16)
Andean Latin America	15 148.2 (11 687.84, 19542.45)	0.85 (0.65, 1.09)
Australasia	505.36 (449.77, 557.99)	0.03 (0.03, 0.04)
Caribbean	5021.30 (3501.38, 9695.61)	1.17 (0.81, 2.26)
Central Asia	14 132.2 (11 883.34, 17006.27)	0.99 (0.83, 1.19)
Central Europe	9763.8 (8367.71, 11251.26)	0.17 (0.15, 0.20)
Central Latin America	15 623.45 (13 507.63, 18131.53)	0.46 (0.40, 0.53)
Central Sub-Saharan Africa	67 751.38 (46 550.28, 97631.13)	10.42 (7.16, 15.01)
East Asia	138 326.13 (112 334.1, 174067.74)	0.48 (0.39, 0.60)
Eastern Europe	66 804.56 (58 987.92, 75382.9)	0.82 (0.72, 0.92)
Eastern Sub-Saharan Africa	117 445.2 (82 039.89, 159189.51)	9.44 (6.60, 12.8)
High-income	36.63 (29.89, 44.57)	0.21 (0.17, 0.25)
High-income Asia Pacific	22 107.47 (18 788.84, 25136.74)	0.25 (0.21, 0.28)
High-income North America	8971.93 (8294.12, 9634.81)	0.03 (0.03, 0.04)
Latin America and Caribbean	97.50 (82.25, 113.66)	0.07 (0.06, 0.08)
North Africa and Middle East	35 043.23 (25 953.24, 51538.87)	0.38 (0.28, 0.56)
Oceania	3129.35 (2507.07, 3881.33)	5.44 (4.36, 6.75)
South Asia	822 493.44 (699 705.48, 1002010.12)	5.70 (4.85, 6.95)
Southeast Asia	370 960.81 (305 231.84, 451691.33)	4.33 (3.56, 5.27)
Southeast Asia, East Asia, and Oceania	91.26 (77.15, 108.15)	1.16 (0.98, 1.38)
Southern Latin America	5519.71 (5125.65, 5945.44)	0.30 (0.28, 0.32)
Southern Sub-Saharan Africa	99 951.75 (87 007.69, 116703.76)	11.59 (10.09, 13.53)
Tropical Latin America	22 016.86 (20 631.81, 23367.54)	0.53 (0.50, 0.56)
Western Europe	14 028.26 (12 594.47, 15365.04)	0.06 (0.05, 0.06)
Western Sub-Saharan Africa	121 065.35 (83 685.33, 165637.07)	5.83 (4.03, 7.97)

Abbreviations: GBD, Global Burden of Disease Study; GDP, Gross Domestic Product; IE, income elasticity; SDI, Sociodemographic index; TB, Tuberculosis; UI, Uncertainty Interval; VLW, Value of Lost Welfare; VSL, Valuation of a Statistical Life.

### 5 SDI Regions

The economic losses associated with TB exhibited considerable variation across different SDI regions. In low-SDI countries, the VLW was estimated at $148.36 billion (95% UI: 107.94, 212.26), accounting for 7.83% of GDP (95% UI: 5.70, 11.20). In low-middle SDI countries, the VLW was $103.72 billion (95% UI: 84.54, 129.36), which represented 5.28% of GDP (95% UI: 4.30, 6.58). For the middle SDI group, the VLW amounted to $49.46 billion (95% UI: 41.63, 59.17), or 2.38% of GDP (95% UI: 2.01, 2.85). In high-middle SDI countries, the VLW was significantly higher at $233.505 billion (95% UI: 196.84, 280.79), constituting 0.46% of GDP (95% UI: 0.39, 0.55). The lowest economic impact was observed in high-SDI countries, with a VLW of $62.38 billion (95% UI: 52.52, 75.23), representing just 0.10% of GDP (95% UI: 0.09, 0.12).

**Figure 1.**
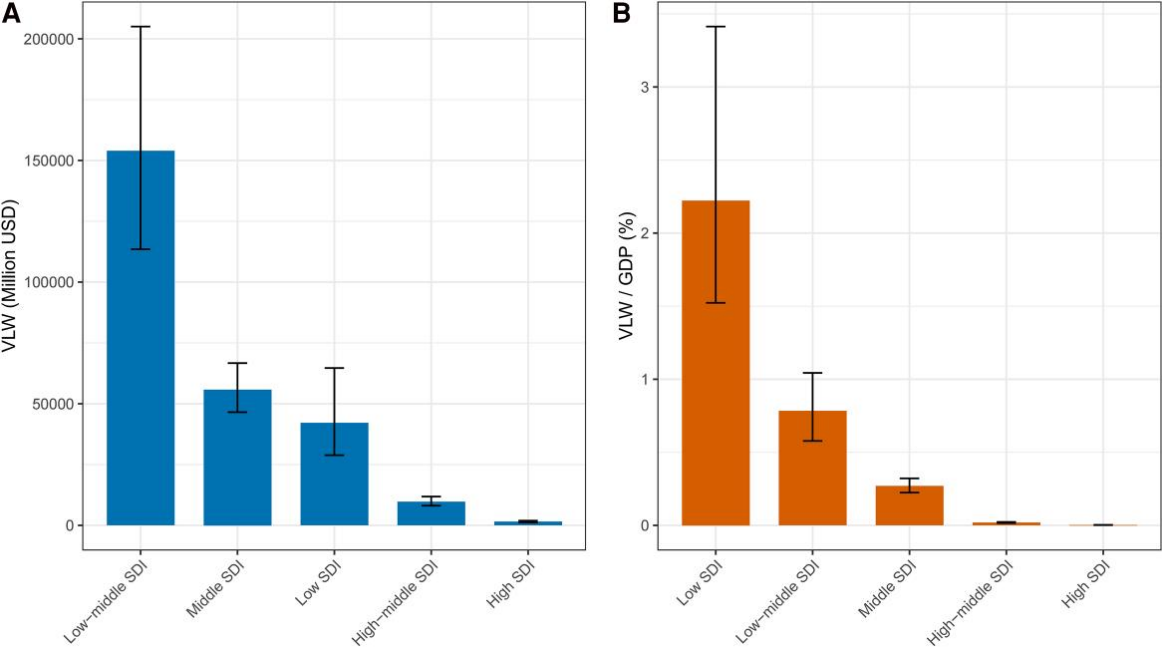
The VLW and the ratio of VLW/GDP for TB across the five SDI Index regions in 2021, generated using an IE of the Value of Statistical Life at 1.00 (*A*: VLW. *B*: VLD/GDP). Abbreviations: GDP, Gross Domestic Product; IE, income elasticity; SDI, Sociodemographic index; TB, Tuberculosis; USD, United States Dollar; VLW, Value of Lost Welfare; VSL, Valuation of a Statistical Life.

These findings highlighted the stark economic disparities in TB's impact across different levels of socio-economic development, underscoring the disproportionate burden borne by lower-income countries (Table 1, Figure 1*A*, *B*).

### 7 GBD Super Regions

In 2021, South Asia experienced the highest overall economic burden due to TB, with a total VLW of $822.493 billion (95% UI: 699.71, 1002.01), representing 5.70% of the region's GDP (95% UI: 4.85, 6.95). This substantial figure underscored the severe economic impact of TB in South Asia, a region with a high disease burden and significant socio-economic challenges. In contrast, Sub-Saharan Africa bore the greatest relative economic loss, with TB-related welfare losses reaching 8.40% of GDP (95% UI: 6.19, 11.16), totaling $406.214 billion (95% UI: 299.28, 539.16). This disproportionate impact highlighted the region's vulnerability to TB, exacerbated by factors such as limited healthcare infrastructure and high levels of poverty. Southeast Asia, East Asia, and Oceania collectively incurred losses of $513.013 billion (95% UI: 420.60, 630.31), amounting to 1.30% of their GDP (95% UI: 1.06, 1.60).

**Figure 2.**
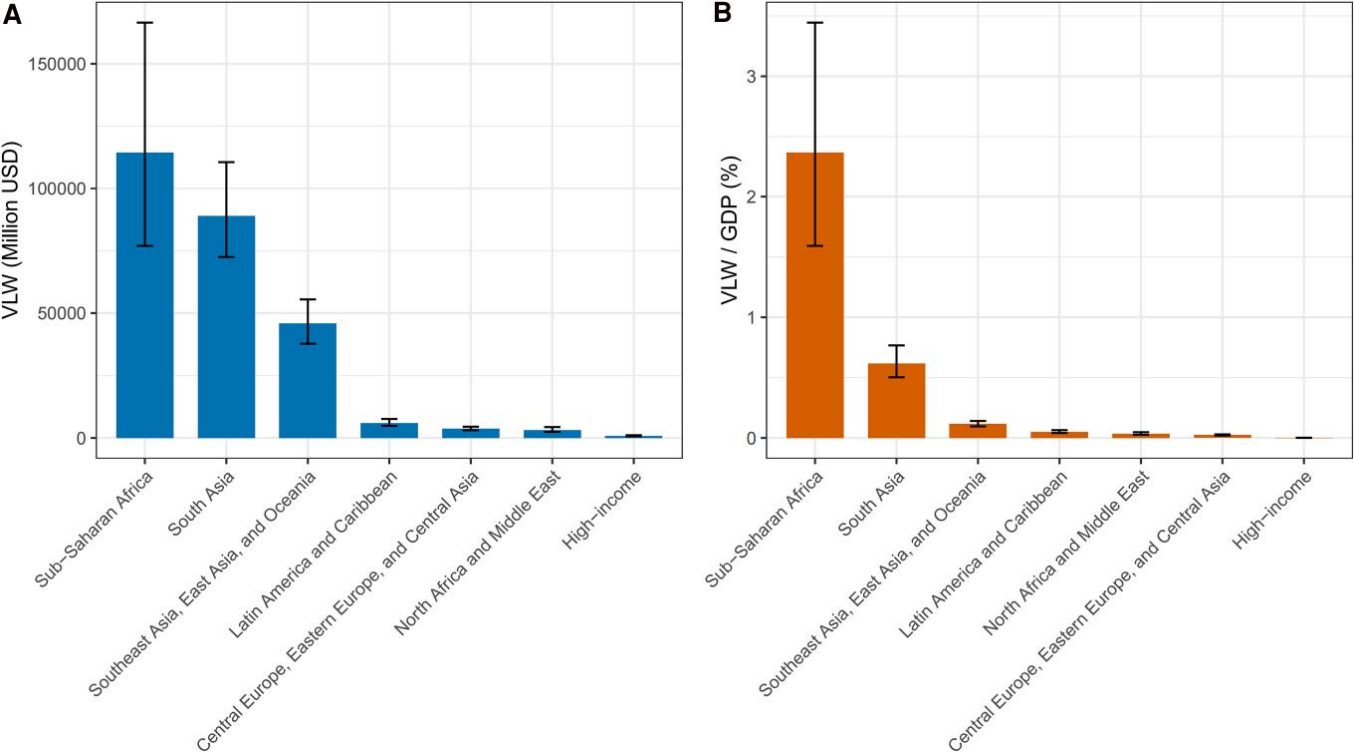
The VLW and the ratio of VLW to VLW/GDP for TB were assessed across the GBD super-regions, using an IE of the VSL set at 1.00 (*A*: VLW. *B*: VLD/GDP). Abbreviations: GBD, Global Burden of Disease Study; GDP, Gross Domestic Product; IE, income elasticity; SDI, Sociodemographic index; TB, Tuberculosis; USD, United States Dollar; VLW, Value of Lost Welfare; VSL, Valuation of a Statistical Life.

In comparison, regions with higher levels of economic development experienced relatively lower welfare losses. Central Europe, Eastern Europe, and Central Asia reported TB-related losses of $90.701 billion (95% UI: 79.24, 103.64), representing 0.60% of their combined GDP (95% UI: 0.52, 0.68). Latin America and the Caribbean faced a VLW of $63.427 billion (95% UI: 54.54, 76.80), corresponding to 0.54% of GDP (95% UI: 0.46, 0.65). North Africa and the Middle East incurred welfare losses totaling $35.043 billion (95% UI: 25.95, 51.54), which represented 0.38% of their GDP (95% UI: 0.28, 0.56). In contrast, high-income countries recorded the lowest economic losses, with a VLW of $45.144 billion (95% UI: 39.71, 50.18), representing only 0.08% of GDP (95% UI: 0.07, 0.09). These findings demonstrated the stark differences in the economic impact of TB across global regions, emphasizing the need for targeted policy interventions in areas most affected by the disease (Table 1, Figure 2*A*, *B*).

### 21 Geographical Regions

In 2021, South Asia and Southeast Asia experienced substantial economic losses due to TB, with the VLW in these regions amounting to $822.493 billion (95% UI: 699.71, 1002.01) and $370.961 billion (95% UI: 305.23, 451.69), respectively. These losses represented 5.70% (95% UI: 4.85, 6.95) and 4.33% (95% UI: 3.56, 5.27) of their respective GDPs, underscoring the significant economic burden of TB in these densely populated regions. Sub-Saharan Africa, particularly the areas south of the Sahara, faced the most severe relative economic impact, with VLW accounting for 11.59% (95% UI: 10.09, 13.53) of the region's GDP, amounting to $99.952 billion (95% UI: 87.01, 116.70). Both Central and Eastern Sub-Saharan Africa saw high economic burdens, with VLW comprising 10.42% (95% UI: 7.16, 15.01) and 9.44% (95% UI: 6.60, 12.80) of GDP, respectively.

**Figure 3.**
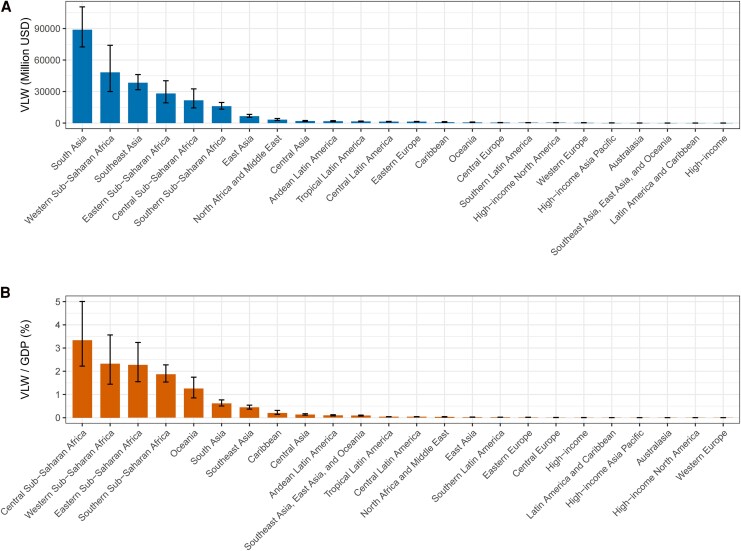
The VLW and the VLW-to-GDP ratio for TB were calculated across the GBD geographic regions, using an income elasticity of the VSL at 1.00 (*A*: VLW. *B*: VLD/GDP). Abbreviations: GBD, Global Burden of Disease Study; GDP, Gross Domestic Product; IE, income elasticity; SDI, Sociodemographic index; TB, Tuberculosis; USD, United States Dollar; VLW, Value of Lost Welfare; VSL, Valuation of a Statistical Life.

In contrast, East Asia recorded a VLW of $138.326 billion (95% UI: 112.33, 174.07), representing 0.48% (95% UI: 0.39, 0.60) of GDP, indicating a relatively lower economic impact. Other regions such as Central Asia (0.99%, 95% UI: 0.83, 1.19), Eastern Europe (0.82%, 95% UI: 0.72, 0.92), and the Caribbean (1.17%, 95% UI: 0.81, 2.26) experienced moderate levels of economic loss due to TB. High-income regions, including North America and Australasia, reported the smallest economic impacts, with VLWs of $8.97 billion (95% UI: 8.29, 9.63) and $0.51 billion (95% UI: 0.50, 0.56), respectively, each accounting for just 0.03% of GDP. These regional disparities in the economic burden of TB emphasized the need for targeted policies and interventions, especially in low- and middle-income countries where the disease placed a disproportionate strain on national economies (Table 1, Figure 3*A*, *B*).

### 204 Countries and Territories

**Figure 4.**
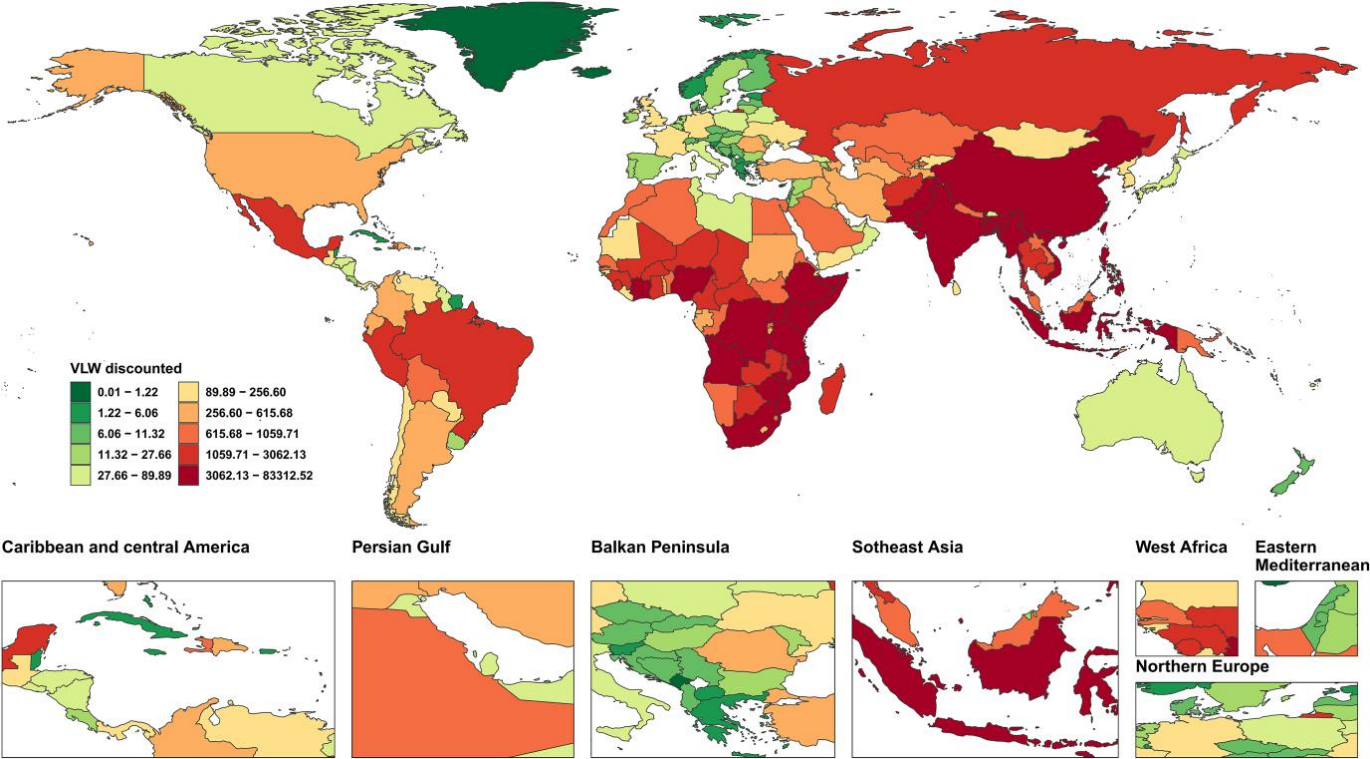
VLW by GBD 204 countries and territories in 2021 for TB, generated using IE of the VSL at 1.00. Abbreviations: GDP, Gross Domestic Product; IE, income elasticity; SDI, Sociodemographic index; TB, Tuberculosis; USD, United States Dollar; VLW, Value of Lost Welfare; VSL, Valuation of a Statistical Life.

The GDP (PPP) data for the following 9 countries and regions were lacking: Cuba, Eritrea, Guam, Monaco, North Korea, Northern Mariana Islands, South Sudan, Venezuela, and Yemen. The missing data were filled using the methods outlined in the methodology. The largest absolute economic losses due to TB were observed in densely populated, high-burden developing countries. India stood at the forefront, with a VLW of $673.98 billion (95% UI: 586.7, 816.10), representing 5.92% (95% UI: 5.15, 7.17) of its GDP. China followed with a VLW of $134.62 billion (95% UI: 109.70, 168.64), accounting for 0.46% (95% UI: 0.38, 0.58) of its GDP. Other nations with significant TB-related economic losses included Indonesia, the Philippines, and South Africa, further highlighting the widespread impact of the disease in these high-burden regions (Figure 4, Supplementary Table 1).

**Figure 5.**
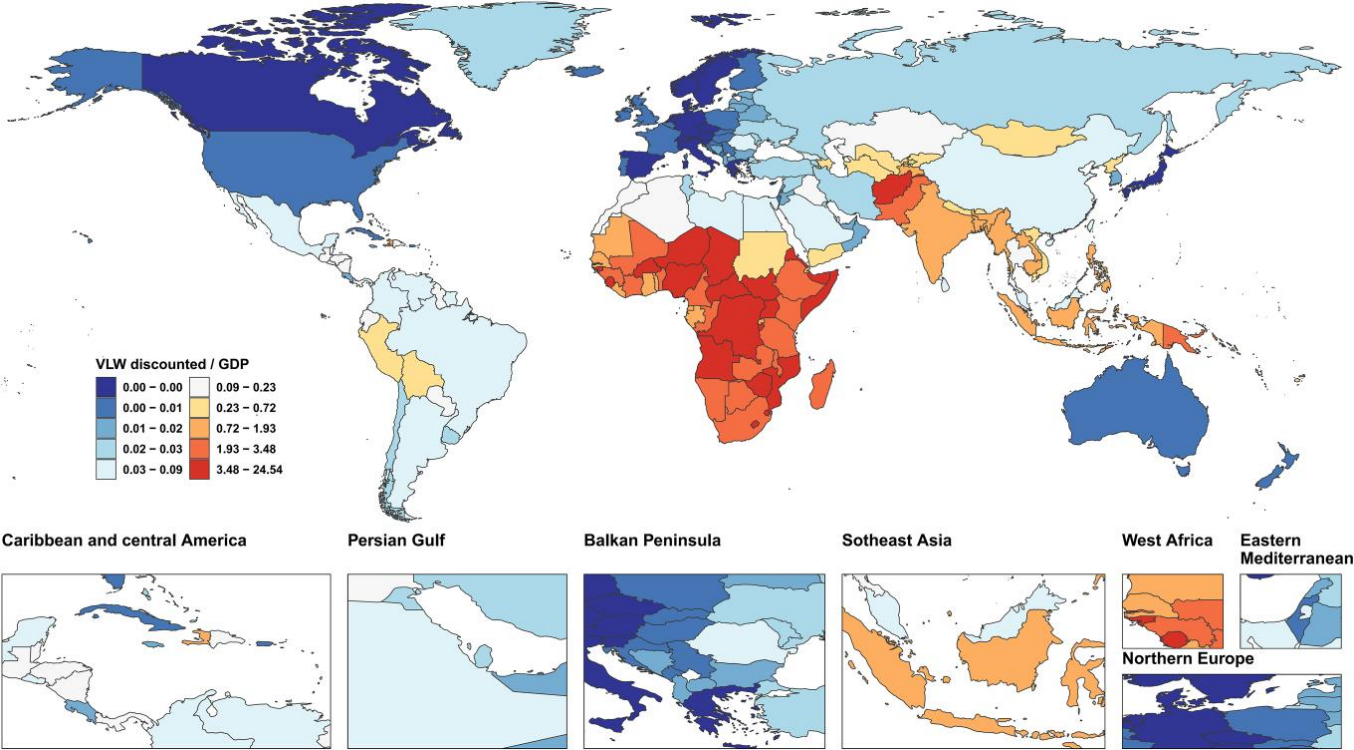
VLW/GDP by GBD 204 countries and territories in 2021 for TB, generated using IE of the VSL at 1.00. Abbreviations: GDP, Gross Domestic Product; IE, income elasticity; SDI, Sociodemographic index; TB, Tuberculosis; USD, United States Dollar; VLW, Value of Lost Welfare; VSL, Valuation of a Statistical Life.

In terms of the VLW/GDP, low-income countries exhibited much higher levels, significantly surpassing the global average of 1.29% (95% UI: 1.05, 1.60). The Central African Republic faced the highest ratio, with TB-related economic losses constituting 51.40% (95% UI: 34.37, 69.47) of GDP, followed by Lesotho (48.64%, 95% UI: 32.83, 62.16) and Zimbabwe (22.10%, 95% UI: 14.42, 29.60) (Figure 5, Supplementary Table 1).

Sub-Saharan Africa and South Asia emerged as the regions with the highest economic losses from TB, demonstrating a strong correlation between the severity of disease burden and economic vulnerability. In stark contrast, high-income nations, such as the United States and the United Kingdom, reported markedly lower VLW-to-GDP ratios, with values below 0.10% (Figures 4 and 5, Supplementary Table 1).

### Sensitivity Analysis

These supplementary tables (Supplementary Tables 2–5) provided detailed insights into the economic impact of TB, allowing for a nuanced understanding of how variations in income elasticity influenced the results at both regional and national levels.

The global economic burden was sensitive to the choice of *ε*. Using *ε* = 0.55, the global VLW was US$5164.46 billion (95% UI: 4163.65, 6520.31), equivalent to 3.36% (95% UI: 2.71, 4.25) of global GDP. In contrast, with *ε* = 1.50, the global VLW was estimated at US$765.42 billion (95% UI: 635.67, 937.62), representing 0.50% (95% UI: 0.41, 0.61) of global GDP.

This pattern was most pronounced across the SDI spectrum. In low-SDI countries, the VLW/GDP ratio was 37.74% (95% UI: 27.31, 54.43) under *ε* = 0.55 but dropped to 1.44% (95% UI: 1.05, 2.04) under *ε* = 1.50. Similarly, in low-middle-SDI countries, the ratio decreased from 14.95% (95% UI: 12.09, 18.70) to 1.68% (95% UI: 1.38, 2.09). The estimates for high-SDI regions remained low and stable, with a VLW/GDP ratio of 0.12% (95% UI: 0.10, 0.14) for *ε* = 0.55 and 0.09% (95% UI: 0.08, 0.11) for *ε* = 1.50.

## DISCUSSION

This study establishes the first robust framework for quantifying the global economic welfare loss attributable to TB, filling a critical gap in existing disease burden research. By employing a standardized VSLY approach, it enables comparable economic loss assessments across countries and provides vital evidence for optimizing resource allocation and TB control strategies in high-burden regions.

This study evaluates the global economic consequences of TB by applying the VLW within the framework of the VSLY, utilizing data from the GBD 2021 and the WHO. The methodology is grounded in the “full income” model proposed by Jamison et al., a framework that has been previously employed by Silva et al. in their analyses of major disease burdens [32]. The findings of this study are in strong agreement with prior authoritative research conducted on both global and regional scales, with high-burden country rankings remaining consistent, supporting the validity and international comparability of the estimates [33]. In contrast, the macroeconomic output loss (VLO) method, developed through collaboration between Klynveld Peat Marwick Goerdeler (KPMG) and WHO, simulates GDP changes in a hypothetical scenario where TB does not exist. This method provides a more conservative estimate of the economic impact of TB. While the two approaches differ in their assumptions and produce slightly varying results, they are highly complementary. The VSLY method emphasizes the broader societal welfare loss, including non-market impacts, while the VLO method is more conservative, focusing solely on observable output losses [34]. Together, these methods provide a comprehensive understanding of the economic burden of TB, capturing both direct and indirect societal costs.

According to projections by the WHO and the Stop TB Partnership, TB is expected to contribute to nearly $1.00 trillion in global economic losses between 2015 and 2030, representing approximately 0.10% of global GDP. These projections reflect a conservative estimate of output losses [33, 35]. However, this study, utilizing GBD 2021 data and the VSLY framework, estimates that TB resulted in $1.9 trillion in global VLW in 2021, equating to 1.29% of global GDP. This figure is substantially higher than those derived from traditional models, highlighting the divergence in the approaches used to assess TB's economic impact [32, 33, 35]. The primary distinction between these estimates lies in the differing perspectives and parameter settings of the methods used. The macroeconomic VLO approach, employed by organizations such as the WHO, focuses on direct economic losses related to labor participation and GDP, omitting the consideration of life years and broader societal welfare. This leads to more conservative estimates. In contrast, the VLW method applied in this study incorporates DALYs into its valuation, offering a more holistic view of the economic consequences of TB on social welfare. It is worth noting that previous research has suggested that DALY valuations within the GBD project may be systematically overestimated, with some diseases and countries’ DALYs being overestimated by factors ranging from 1.5 to 17 times [36]. This overestimation could further inflate the VLW estimates presented in this study. Therefore, while the results from this study exceed those from traditional models, they underscore essential differences in estimation techniques, data sources, and analytical frameworks. These differences highlight the importance of understanding the assumptions underlying each model and the contextual relevance of economic burden metrics when informing policy decisions.

The economic impact of TB is marked by significant regional disparities, with the highest burdens falling on low-income and lower-middle-income countries, especially in Southeast Asia and Africa. Previous research has indicated that from 2000 to 2015, Southeast Asia experienced the largest cumulative GDP loss due to TB, followed by Africa and the Western Pacific region, with Africa projected to bear nearly one-third of the global economic losses associated with the disease [32, 33]. Smaller economies with high TB mortality rates, such as Cambodia, Mozambique, and Lesotho, are predicted to endure cumulative economic losses exceeding 3% of their annual GDP between 2015 and 2030. In approximately 15 countries in sub-Saharan Africa, TB-related welfare losses could surpass 10% of annual GDP [32, 33, 35]. This study further confirms and quantifies these disparities, revealing that the VLW-to-GDP ratio in several African nations ranges from 10% to 50%, which is markedly higher than in other regions. In some cases, these figures exceed previous estimates. This difference may stem from the higher DALY estimates in the GBD data used in this study, as well as the VSLY methodology's inclusion of non-market welfare losses in the valuation of life years. By contrast, output loss models, such as those employed by organizations like WHO and KPMG, take a more conservative approach, focusing exclusively on direct GDP losses and excluding the indirect costs associated with the loss of life value and broader social welfare. This distinction results in systematic differences in the scale of economic valuation between the two methodologies.The findings highlight the critical need for region-specific strategies and greater investment in TB control, particularly in regions most affected by the disease [32, 33, 35].

Despite minor differences in the methodologies used across various studies, there is significant consistency in identifying the countries that bear the greatest economic burden from TB. These countries are typically characterized by high TB incidence, large populations, and significant economic activity, and they consistently rank among the top for economic losses [33, 35]. This study's country rankings align closely with those found in previous research. India, which has the highest number of TB cases and deaths, consistently emerges as the country with the most substantial economic loss. China, driven by its vast population and large economy, usually ranks second or third in terms of economic burden [33, 35]. For instance, the KPMG report identifies the top ten countries with the highest GDP losses due to TB, which include India, Indonesia, China, Russia, Nigeria, South Africa, Japan, Pakistan, Brazil, and South Korea. With the exception of a few high-income nations, the majority of these countries are developing economies that face considerable TB burdens. The results from this study closely align with these rankings, providing further validation for the robustness and comparability of the economic loss estimates. This consistency underscores the significance of TB as a major global health and economic issue, particularly in regions with high disease prevalence and large populations, highlighting the critical need for targeted interventions and investment in TB control [33, 35].

The study found that in low-income countries, the economic burden of TB represents a disproportionately high share of national GDP, with some regions of Sub-Saharan Africa exceeding 5%. This is primarily due to the high TB-related DALY burden across all age groups in these middle- and low-income countries, particularly among younger populations and adults with longer remaining life expectancy. Additionally, within the VSLY framework, deaths and severe disabilities occurring at younger ages lead to greater lifetime welfare losses, significantly amplifying the economic impact per case. Despite the absolute size of VLW being relatively modest when measured in US dollars, the ratio of VLW to GDP remains high due to both the low total GDP and per capita GDP in these countries. Therefore, a ratio exceeding 5% does not mean that 5%–20% of these countries’ 2021 total GDP was directly lost, but rather indicates that the discounted lifetime welfare losses attributable to TB are equivalent to a substantial proportion of the current national economic output, revealing a profound structural vulnerability at the macroeconomic level.

Several limitations should be considered. Firstly, although the research is based on the GBD 2021 database, many low- and middle-income countries and regions face challenges such as incomplete TB surveillance systems, shortages of healthcare personnel, weak healthcare infrastructure, and limited macroeconomic statistical capacity. These issues constrain the completeness and quality of TB burden and related economic data, potentially affecting the stability and reliability of the model estimates [12, 13, 27]. Secondly, the lack of macroeconomic monitoring data in low-income regions further increases the uncertainty of the economic assessment results [20, 23]. Thirdly, the VSLY methodology depends on generalized assumptions, such as the use of uniform VSL factors and income elasticity values. These assumptions may not fully capture the cultural, socio-economic, and institutional differences that exist across countries, potentially reducing the method's relevance and applicability in certain contexts [20, 23]. This study primarily focuses on economic losses associated with mortality and non-fatal impacts, such as healthcare costs, treatment expenses, and the long-term socio-economic consequences of TB. However, it does not account for the impact of drug-resistant TB and HIV-TB co-infection on the economic burden of TB [15, 37]. These factors are likely to amplify the economic burden of TB, and their omission may lead to an underestimation of the true economic costs associated with the disease. To mitigate these limitations, future research should incorporate more detailed, country-specific cost analyses and utilize advanced macroeconomic modeling techniques to provide a more comprehensive assessment of TB's economic impact. Such analyses would be crucial for informing policy decisions and guiding fiscal planning in TB control and prevention efforts.

## CONCLUSIONS

The study provided critical data to inform policy decisions and highlighted TB's dual challenge of worsening health inequities and causing significant human capital loss, threatening global economic stability. In low-SDI regions, such as sub-Saharan Africa and South Asia, TB-related VLW exceeded 10% of GDP, with some countries facing losses as high as 50%. This stark disparity emphasized the severe economic strain TB placed on vulnerable economies. The findings called for the urgent incorporation of TB control measures into global health and economic policies, with increased financial support and multilateral cooperation, particularly in high-burden regions, to mitigate its impact and support sustainable development goals.

## Supplementary Material

ofag014_Supplementary_Data

## Supplementary Data

Supplementary materials are available at *Open Forum Infectious Diseases* online. Consisting of data provided by the authors to benefit the reader, the posted materials are not copyedited and are the sole responsibility of the authors, so questions or comments should be addressed to the corresponding author.
