# Supplementary material for: The Global Economic Burden of Tuberculosis: Regional Disparities and Implications
Source: Open Forum Infect Dis. 2026 Jan 29;13(1):ofag014. doi: 10.1093/ofid/ofag014 (PMC12852480; doi:10.1093/ofid/ofag014)
Supplement: ofag014_Supplementary_Data [file ofag014_supplementary_data.docx]

**Supplementary**

**The Global Economic Burden of Tuberculosis: Regional Disparities and Implications**

Health Economic Analysis Plan

Table 1. VLW and VLW/GDP by 204countries or territories 2021 for TB, Generated Using Income Elasticity (IE) of the VSL at 1.00.

Table 2. VLW and VLW/GDP by 204countries or territories 2021 for TB, Generated Using Income Elasticity (IE) of the VSL at 0.55.

Table 3. VLW and VLW/GDP by 204countries or territories 2021 for TB, Generated Using Income Elasticity (IE) of the VSL at 1.50.

Table 4. VLW and VLW/GDP by GBD regions in 2021 for TB, Generated Using Income Elasticity (IE) of the VSL at 0.55.

Table 5. VLW and VLW/GDP by GBD regions in 2021 for TB, Generated Using Income Elasticity (IE) of the VSL at 1.50.

Health Economic Analysis Plan

Study Title:

Economic Burden of Tuberculosis: A Global Health Economic Evaluation Using the Value of Statistical Life Year Framework (VSLY)

1. Study Objective

This study aims to evaluate the economic burden of tuberculosis (TB) globally, utilizing the Value of a Statistical Life Year (VSLY) framework. The goal is to estimate the Value of Lost Welfare (VLW) attributable to TB in terms of monetary welfare losses, incorporating data from the Global Burden of Disease (GBD) 2021 and the World Bank. The study assesses how the economic impact varies across countries with differing socio-demographic indexes (SDI) and income levels, and to identify high-burden regions where targeted investments in TB control are needed.

2. Study Population

This economic evaluation utilizes data from the Global Burden of Disease (GBD) 2021 study, encompassing 204 countries and territories globally. The study includes individuals of all ages, across all socio-economic backgrounds, and reflects the health burden due to TB for different population groups based on factors such as age, gender, and socio-demographic index (SDI). The study uses DALY (Disability-Adjusted Life Years) as the main health measure to quantify both the loss of life and life lived with disability caused by TB.

3. Study Perspective

The perspective of this economic analysis is societal, considering all direct and indirect costs associated with TB-related mortality and morbidity. This includes both market (such as lost income due to illness, healthcare costs) and non-market (such as quality of life and productivity loss) impacts. By adopting this perspective, the study accounts for the broader societal welfare loss, emphasizing the economic burden not just on the healthcare system but also on national economies and individuals’ livelihoods.

4. Time Horizon

The time horizon for this analysis is 1 year (2021), consistent with the most recent available data from GBD 2021 and the World Bank. The study quantifies the welfare losses due to TB for the year 2021. Sensitivity analyses are conducted to explore how results change over different time horizons, including longer-term projections based on TB incidence rates and economic growth.

5. Discount Rate

A 3% annual discount rate is applied to convert future welfare losses into present value, consistent with established health economics practices. This rate reflects the time preference for current versus future consumption. This is in line with previous studies assessing the economic burden of global diseases and allows for comparability across studies.

6. Selection of Outcomes

The primary outcome for this study is the Value of Lost Welfare (VLW), which is calculated by monetizing the Disability-Adjusted Life Years (DALYs) attributed to TB. DALYs capture the combined loss of life and the burden of living with TB-related disabilities. The Value of a Statistical Life Year (VSLY) is used as the basis for monetizing DALYs. This method integrates willingness-to-pay (WTP) measures, adjusted for income elasticity (IE), which is estimated differently across countries with varying levels of economic development.

The specific outcomes measured include:

Economic welfare loss due to TB (VLW)

DALYs due to TB mortality and morbidity

VSLY for each country, adjusted for income elasticity

7. Measurement of Outcomes

DALYs: DALYs are sourced from the GBD 2021 study, which provides a global and regional estimate of disease burden, including mortality and morbidity for all diseases, including TB.

VSLY: The VSLY is estimated by translating DALYs into economic terms, using the standard VSL for the United States in 2021 as the baseline, adjusted for country-specific income elasticity (IE). The VSLY estimates the monetary value of each year of life lost due to TB.

VLW: The VLW is the sum of the VSLY values across all countries and regions, capturing the total welfare loss attributed to TB globally.

8. Valuation of Outcomes

The value of the outcomes is based on the VSLY framework, which incorporates the Value of a Statistical Life (VSL) for 2021. The VSL for the United States is used as the baseline (obtained from the US Department of Transportation), and it is adjusted for the income elasticity of VSL (IE), varying across countries depending on GDP per capita. The IE is used to account for income-driven differences in willingness to pay for life-saving interventions.

9. Measurement and Valuation of Resources and Costs

Gross Domestic Product (GDP) per capita data, adjusted for purchasing power parity (PPP), is retrieved from the World Bank’s World Development Indicators database. These data are used to adjust for differences in economic capacity across countries and calculate the economic loss associated with TB for each country and region.

Cost Valuation: Direct costs (e.g., medical expenses) and indirect costs (e.g., productivity losses) are quantified by integrating DALYs with country-specific economic data.

10. Rationale and Description of Model

This study utilizes a full-income model, which has been previously applied in global economic burden studies for diseases like cancer and stroke. The model integrates health data (DALYs) with macroeconomic data (GDP and VSLY) to provide a comprehensive assessment of TB's economic impact.

11. Analytics and Assumptions

Monte Carlo simulations will be employed to capture uncertainty across key inputs, including DALY estimates, VSLY values, and GDP data.

Sensitivity analysis will be performed to evaluate the impact of varying income elasticity values (from 0.55 to 1.50) on the economic burden estimates. This ensures that the results remain robust across different income contexts and provides policymakers with an understanding of the potential range of outcomes.

12. Characterizing Uncertainty

Uncertainty in the analysis will be characterized using 95% uncertainty intervals (UI) derived from the GBD 2021 data for DALYs and from the statistical distribution of the VSLY model. The impact of different assumptions on the economic estimates will also be evaluated through sensitivity analyses.

13. Approach to Engagement with Stakeholders

Stakeholder engagement will involve consultations with global health policymakers, particularly those in low- and middle-income countries (LMICs), which bear the highest economic burdens from TB. The study’s findings will be shared with organizations like the World Health Organization (WHO), Global Fund, and national ministries of health to inform TB control policies and strategies.

14. Ethical Considerations

As the study relies on publicly available data from the GBD 2021 and the World Bank, no ethical approval is required for the economic analysis. All data processing and analysis comply with standard guidelines for secondary data use in health economics research.

**Methodology Section**

#### ****(This version provides a detailed, transparent, and reproducible methodology, aligning with the CHEERS and EQUATOR guidelines)****

**Study Design and Analytic Framework**

This study constitutes a population-based, retrospective global assessment of the economic burden of disease. We employed the Value of a Statistical Life Year (VSLY)framework to monetize the health losses attributable to tuberculosis (TB) into a Value of Lost Welfare (VLW). This framework, grounded in the "full-income" approach, translates the total health loss measured in disability-adjusted life years (DALYs) into a comparable economic metric, aiming to evaluate the macroeconomic welfare loss from a societal willingness-to-pay perspective.

**Study Perspective and Target Population**

The analysis adopted a societal perspective, aiming to capture the full societal resource consumption and welfare loss due to TB. The study encompassed 204 countries and territories globally. The target population was the entire population within these regions for the year 2021. Analyses were stratified by age (following GBD standard age groups), sex, and country to investigate the demographic and geographical distribution of the disease burden.

**Data Sources**

The research integrated several authoritative global databases:

1. Health Outcome Data: Data on TB-related disability-adjusted life years (DALYs), life tables (for calculating life expectancy), and population demographics were sourced from the Global Burden of Disease Study 2021 (GBD 2021)**, coordinated by the Institute for Health Metrics and Evaluation (IHME) at the University of Washington. This dataset provides standardized estimates of disease burden, disaggregated by age, sex, country, and territory, accompanied by 95% uncertainty intervals (UI).

2. Economic Data: Country-specific purchasing power parity (PPP)-adjusted Gross Domestic Product per capita (GDP per capita, PPP) data (in constant 2017 international dollars) were obtained from the **World Bank's World Development Indicators database Aggregate GDP figures for global and regional levels were also sourced from here.

3. Core Valuation Parameter: The baseline Value of a Statistical Life (VSL)** was obtained from the official 2021 estimates published by the **United States Department of Transportation (USDOT)**. This VSL valuation, derived from wage-risk studies in the US labor market, serves as an internationally recognized foundational parameter for deriving VSLY.

All monetary values were standardized to the **2021 fiscal year** to ensure consistency across data sources and time dimensions.

**Measurement and Valuation of Health Outcomes: Calculation of VSLY and VLW**

The core of this study was the monetization of TB-related DALY losses. The specific steps were as follows:

1. Calculation of Country-Specific Value of a Statistical Life

First, the US benchmark VSL was adjusted to each country using an income elasticity function. The formula is:

`VSL_i = VSL_USA * (GDP_pc_i / GDP_pc_USA)^ε`

Where `VSL_USA` is the US benchmark VSL, `GDP_pc_i` and `GDP_pc_USA` are the PPP-adjusted per capita GDP of the specific country and the USA, respectively, and `ε` is the **income elasticity**, reflecting how willingness-to-pay changes with income. The base-case analysis used `ε = 1.00`.

2. Calculation of the Value of a Statistical Life Year

The country-specific VSL was then allocated over an individual's remaining life expectancy, accounting for time preference (discounting) and the relative weight of life value at different ages (age-weighting). The formula is:

`VSLY_i,a = [VSL_i * γ(a)] / Σ_{t=0}^{LE_i,a-1} [ (1+δ)^{-t} ]`

Here, `γ(a)` represents the age-weighting function (sourced from GBD studies), `LE_i,a` is the remaining life expectancy for age group *a* in country *i*, and `δ` is the annual discount rate, set at **3.0%** for the base-case analysis.

1. Calculation of the Tuberculosis Value of Lost Welfare

Finally, the TB-related DALY losses for each age group in each country were multiplied by the corresponding VSLY and summed to obtain the total VLW.

`VLW_global = Σ_i Σ_a (DALYs_i,a * VSLY_i,a)`

Additionally, we calculated VLW as a percentage of the country's or region's GDP (VLW/GDP%), to measure the relative economic burden.

**Analytical Methods and Key Assumptions**

Sensitivity Analysis: To examine the impact of uncertainty in the core parameter (income elasticity ε) on the results, we conducted extensive sensitivity analyses, recalculating global and regional VLW using `ε = 0.55` (less income-sensitive willingness-to-pay) and `ε = 1.50` (highly income-sensitive willingness-to-pay).

Uncertainty Propagation: The DALY estimates from GBD 2021 inherently include a 95% uncertainty interval. We propagated this uncertainty into the final VLW estimates using a Monte Carlo simulation approach, reporting the 95% UI for both VLW and its proportion of GDP.

Missing Data Handling: For a small number of countries/territories with missing 2021 GDP (PPP) data, we employed the following imputation strategy: (1) priority was given to interpolation using the median of data from the preceding and following 3-5 years; (2) if no subsequent data were available, the most recent historical data were used; (3) for non-sovereign regions, data from sovereign countries at the same Socio-demographic Index (SDI) level were borrowed, or the median of neighboring countries at the same SDI level was applied. All computations and statistical analyses were performed in the **RStudio** environment.

**Study Reporting Standards**

The design, conduct, and reporting of this study strictly adhered to the **Consolidated Health Economic Evaluation Reporting Standards 2022 (CHEERS 2022)** checklist to ensure methodological rigor and transparency of results (see Supplementary Materials for the completed checklist).

#### ****Ethical Considerations****

This study utilized publicly available global datasets, and all data were anonymized. As no individual-level data were collected, the study did not require institutional review board (IRB) approval. The secondary data used comply with all relevant ethical guidelines, and the analysis was conducted in accordance with ethical research practices.

Table 1. VLW and VLW/GDP by 204countries or territories 2021 for TB, Generated Using Income Elasticity (IE) of the VSL at 1.00.

| Location_id | Location name | VSLY | VLW region  (millions) | VLW region  (millions)  95%UI lower | VLW region (millions)  95%UI upper | VLW/GDP (%) | VLW/GDP(%) 95%UI lower | VLW/GDP (%) 95%UI upper |
| --- | --- | --- | --- | --- | --- | --- | --- | --- |
| 6 | China | 97871.88 | 134623.75 | 109696.73 | 168640.32 | 0.46 | 0.38 | 0.58 |
| 7 | Democratic People's Republic of Korea | 5542.92 | 1988.38 | 1425.12 | 2847.88 | 6.85 | 4.91 | 9.81 |
| 10 | Cambodia | 34104.43 | 8653.23 | 5935.03 | 12484.46 | 8.16 | 5.59 | 11.77 |
| 11 | Indonesia | 68124.12 | 215418.00 | 179722.09 | 257151.40 | 6.05 | 5.05 | 7.23 |
| 12 | Lao People's Democratic Republic | 44218.39 | 3172.63 | 1980.69 | 4389.96 | 5.32 | 3.32 | 7.36 |
| 13 | Malaysia | 153197.98 | 10751.44 | 9395.27 | 12663.42 | 1.13 | 0.99 | 1.33 |
| 14 | Maldives | 90367.46 | 71.20 | 57.76 | 87.66 | 0.72 | 0.59 | 0.89 |
| 15 | Myanmar | 28575.45 | 16757.69 | 12888.73 | 22436.58 | 5.74 | 4.41 | 7.68 |
| 16 | Philippines | 48404.34 | 52797.79 | 44953.82 | 60944.10 | 5.26 | 4.48 | 6.08 |
| 17 | Sri Lanka | 71030.02 | 2501.89 | 1816.99 | 3441.60 | 0.78 | 0.57 | 1.08 |
| 18 | Thailand | 100047.57 | 25385.28 | 20536.73 | 31632.59 | 1.88 | 1.52 | 2.34 |
| 19 | Timor-Leste | 37315.32 | 876.72 | 640.23 | 1229.08 | 9.19 | 6.71 | 12.88 |
| 20 | Socialist Republic of Viet Nam | 60388.73 | 36956.88 | 29017.22 | 48533.93 | 3.06 | 2.40 | 4.02 |
| 22 | Fiji | 60360.65 | 114.51 | 90.40 | 145.40 | 1.16 | 0.92 | 1.47 |
| 23 | Kiribati | 17827.07 | 62.48 | 47.29 | 84.28 | 16.91 | 12.80 | 22.81 |
| 24 | Marshall Islands | 38652.08 | 51.66 | 33.95 | 72.06 | 13.69 | 9.00 | 19.09 |
| 25 | Federated States of Micronesia | 21862.14 | 11.19 | 8.40 | 14.81 | 2.78 | 2.09 | 3.68 |
| 26 | Papua New Guinea | 23387.33 | 2782.32 | 2247.16 | 3423.01 | 6.69 | 5.40 | 8.23 |
| 27 | Samoa | 32996.99 | 18.84 | 14.69 | 24.63 | 1.42 | 1.11 | 1.86 |
| 28 | Solomon Islands | 14234.74 | 50.90 | 39.79 | 66.25 | 2.96 | 2.31 | 3.85 |
| 29 | Tonga | 36110.90 | 6.84 | 5.33 | 8.97 | 0.91 | 0.71 | 1.20 |
| 30 | Vanuatu | 17728.32 | 26.07 | 16.78 | 36.23 | 2.66 | 1.71 | 3.69 |
| 33 | Armenia | 79843.62 | 136.78 | 119.79 | 155.53 | 0.29 | 0.25 | 0.33 |
| 34 | Azerbaijan | 106867.07 | 2463.89 | 1844.70 | 3535.49 | 1.17 | 0.87 | 1.67 |
| 35 | Georgia | 99299.30 | 447.45 | 396.55 | 501.98 | 0.66 | 0.58 | 0.74 |
| 36 | Kazakhstan | 177570.47 | 4927.39 | 4396.18 | 5452.81 | 0.79 | 0.70 | 0.87 |
| 37 | Kyrgyzstan | 29721.92 | 481.17 | 401.95 | 566.89 | 1.22 | 1.02 | 1.44 |
| 38 | Mongolia | 79377.32 | 1714.00 | 1212.25 | 2579.54 | 3.47 | 2.46 | 5.23 |
| 39 | Tajikistan | 21441.33 | 842.72 | 628.09 | 1102.31 | 2.08 | 1.55 | 2.72 |
| 40 | Turkmenistan | 90180.71 | 1713.79 | 1375.72 | 2138.15 | 2.02 | 1.62 | 2.52 |
| 41 | Uzbekistan | 47905.50 | 3119.00 | 2720.35 | 3553.10 | 0.99 | 0.86 | 1.12 |
| 43 | Albania | 79890.97 | 78.20 | 55.12 | 123.11 | 0.18 | 0.13 | 0.29 |
| 44 | Bosnia and Herzegovina | 92028.81 | 275.35 | 217.22 | 342.73 | 0.46 | 0.36 | 0.57 |
| 45 | Bulgaria | 157209.15 | 436.68 | 380.29 | 499.41 | 0.22 | 0.19 | 0.25 |
| 46 | Croatia | 181130.94 | 254.73 | 221.23 | 288.01 | 0.16 | 0.14 | 0.19 |
| 47 | Czech Republic | 234639.91 | 237.02 | 208.40 | 275.20 | 0.05 | 0.04 | 0.05 |
| 48 | Hungary | 194301.75 | 373.98 | 327.26 | 421.77 | 0.10 | 0.09 | 0.11 |
| 49 | North Macedonia | 116162.91 | 122.45 | 91.04 | 166.22 | 0.25 | 0.19 | 0.34 |
| 50 | Montenegro | 124193.50 | 25.12 | 19.53 | 32.65 | 0.17 | 0.13 | 0.22 |
| 51 | Poland | 205356.48 | 2918.52 | 2630.45 | 3199.12 | 0.19 | 0.17 | 0.20 |
| 52 | Romania | 194108.08 | 5636.00 | 4932.19 | 6297.03 | 0.79 | 0.69 | 0.88 |
| 53 | Serbia | 118579.55 | 399.06 | 316.84 | 492.93 | 0.19 | 0.15 | 0.24 |
| 54 | Slovakia | 193725.50 | 206.74 | 166.38 | 255.15 | 0.10 | 0.08 | 0.12 |
| 55 | Slovenia | 216972.17 | 47.95 | 41.68 | 55.26 | 0.05 | 0.04 | 0.06 |
| 57 | Belarus | 146325.52 | 1351.65 | 1091.80 | 1643.02 | 0.52 | 0.42 | 0.64 |
| 58 | Estonia | 218617.01 | 150.04 | 131.32 | 169.83 | 0.26 | 0.23 | 0.29 |
| 59 | Latvia | 190326.39 | 304.76 | 269.22 | 337.63 | 0.44 | 0.39 | 0.49 |
| 60 | Lithuania | 235703.56 | 855.53 | 755.78 | 952.47 | 0.68 | 0.60 | 0.76 |
| 61 | Republic of Moldova | 81700.75 | 461.87 | 411.52 | 517.10 | 0.82 | 0.73 | 0.92 |
| 62 | Russian Federation | 210310.59 | 48575.85 | 44685.93 | 52640.80 | 0.86 | 0.79 | 0.93 |
| 63 | Ukraine | 95312.28 | 9187.59 | 6565.20 | 12339.90 | 1.20 | 0.85 | 1.61 |
| 66 | Brunei Darussalam | 384584.41 | 191.16 | 162.04 | 225.79 | 0.54 | 0.46 | 0.64 |
| 67 | Japan | 197338.39 | 10310.34 | 8620.74 | 11367.92 | 0.18 | 0.15 | 0.20 |
| 68 | Republic of Korea | 220967.78 | 11009.87 | 9466.82 | 12886.48 | 0.44 | 0.38 | 0.52 |
| 69 | Singapore | 578425.90 | 787.26 | 701.27 | 882.34 | 0.10 | 0.09 | 0.12 |
| 71 | Australia | 270263.31 | 457.33 | 409.95 | 500.68 | 0.03 | 0.03 | 0.03 |
| 72 | New Zealand | 224718.22 | 48.03 | 39.82 | 57.31 | 0.02 | 0.02 | 0.02 |
| 74 | Andorra | 274516.92 | 0.67 | 0.48 | 0.91 | 0.01 | 0.01 | 0.02 |
| 75 | Austria | 294721.81 | 336.98 | 304.23 | 368.53 | 0.06 | 0.05 | 0.07 |
| 76 | Belgium | 285321.22 | 361.43 | 321.42 | 400.05 | 0.05 | 0.05 | 0.06 |
| 77 | Cyprus | 228320.15 | 32.41 | 27.50 | 38.99 | 0.05 | 0.04 | 0.06 |
| 78 | Denmark | 326206.21 | 149.11 | 134.31 | 166.08 | 0.04 | 0.03 | 0.04 |
| 79 | Finland | 265509.46 | 244.28 | 217.28 | 267.50 | 0.08 | 0.07 | 0.09 |
| 80 | France | 244847.65 | 2957.86 | 2607.89 | 3236.13 | 0.08 | 0.07 | 0.09 |
| 81 | Germany | 296879.94 | 2937.56 | 2626.08 | 3246.61 | 0.06 | 0.05 | 0.06 |
| 82 | Greece | 160269.11 | 323.55 | 295.34 | 350.25 | 0.09 | 0.09 | 0.10 |
| 83 | Iceland | 281919.74 | 9.27 | 8.17 | 10.27 | 0.04 | 0.04 | 0.05 |
| 84 | Ireland | 552081.46 | 278.83 | 247.89 | 309.11 | 0.05 | 0.04 | 0.05 |
| 85 | Israel | 211524.99 | 138.51 | 122.28 | 152.34 | 0.03 | 0.03 | 0.03 |
| 86 | Italy | 231182.21 | 1350.54 | 1212.07 | 1464.27 | 0.05 | 0.04 | 0.05 |
| 87 | Luxembourg | 626980.71 | 21.41 | 18.82 | 24.54 | 0.02 | 0.02 | 0.03 |
| 88 | Malta | 262025.72 | 5.99 | 5.20 | 7.00 | 0.02 | 0.02 | 0.03 |
| 89 | Netherlands | 320984.84 | 500.12 | 448.77 | 551.23 | 0.04 | 0.04 | 0.05 |
| 90 | Norway | 409851.15 | 291.93 | 261.57 | 317.30 | 0.06 | 0.05 | 0.07 |
| 91 | Portugal | 182196.22 | 794.02 | 729.02 | 869.16 | 0.19 | 0.18 | 0.21 |
| 92 | Spain | 204440.65 | 1218.76 | 1096.20 | 1339.29 | 0.06 | 0.05 | 0.07 |
| 93 | Sweden | 287818.09 | 265.70 | 229.61 | 299.46 | 0.04 | 0.04 | 0.05 |
| 94 | Switzerland | 369532.94 | 234.71 | 208.01 | 261.73 | 0.03 | 0.03 | 0.04 |
| 95 | United Kingdom of Great Britain and Northern Ireland | 243808.65 | 1712.40 | 1594.13 | 1835.69 | 0.05 | 0.05 | 0.05 |
| 97 | Argentina | 131252.16 | 3462.28 | 3225.90 | 3714.49 | 0.29 | 0.27 | 0.31 |
| 98 | Chile | 141559.14 | 1801.42 | 1662.96 | 1952.50 | 0.33 | 0.30 | 0.36 |
| 99 | Uruguay | 147739.94 | 256.01 | 236.79 | 278.45 | 0.26 | 0.24 | 0.28 |
| 101 | Canada | 265998.57 | 705.73 | 641.71 | 775.01 | 0.03 | 0.03 | 0.04 |
| 102 | United States of America | 363927.34 | 8266.19 | 7652.41 | 8859.80 | 0.03 | 0.03 | 0.04 |
| 105 | Antigua and Barbuda | 129972.03 | 1.94 | 1.65 | 2.30 | 0.08 | 0.07 | 0.10 |
| 106 | Commonwealth of the Bahamas | 158028.97 | 37.56 | 30.56 | 46.53 | 0.33 | 0.27 | 0.41 |
| 107 | Barbados | 78084.36 | 4.24 | 3.43 | 5.25 | 0.09 | 0.07 | 0.11 |
| 108 | Belize | 60323.24 | 30.05 | 26.58 | 33.56 | 0.60 | 0.53 | 0.67 |
| 109 | Cuba | 21714.79 | 27.89 | 23.80 | 32.86 | 0.06 | 0.05 | 0.07 |
| 110 | Dominica | 83752.70 | 6.74 | 5.18 | 8.87 | 0.64 | 0.50 | 0.85 |
| 111 | Dominican Republic | 112750.46 | 3236.30 | 2372.28 | 5023.32 | 1.34 | 0.98 | 2.08 |
| 112 | Grenada | 82359.55 | 1.49 | 1.24 | 1.73 | 0.09 | 0.08 | 0.11 |
| 113 | Guyana | 134884.79 | 281.74 | 218.40 | 360.33 | 1.61 | 1.25 | 2.06 |
| 114 | Haiti | 19804.14 | 1555.11 | 944.32 | 4386.96 | 3.86 | 2.34 | 10.88 |
| 115 | Jamaica | 48673.79 | 24.44 | 19.29 | 30.62 | 0.09 | 0.07 | 0.11 |
| 116 | Saint Lucia | 99590.31 | 13.13 | 10.98 | 15.63 | 0.39 | 0.32 | 0.46 |
| 117 | Saint Vincent and the Grenadines | 83420.58 | 5.55 | 4.92 | 6.31 | 0.31 | 0.27 | 0.35 |
| 118 | Suriname | 99599.68 | 40.21 | 30.84 | 50.51 | 0.38 | 0.29 | 0.47 |
| 119 | Trinidad and Tobago | 164875.35 | 101.45 | 79.62 | 128.05 | 0.24 | 0.19 | 0.30 |
| 121 | Plurinational State of Bolivia | 53626.93 | 3034.70 | 2119.11 | 4125.79 | 2.72 | 1.90 | 3.70 |
| 122 | Ecuador | 69212.20 | 1360.68 | 1125.54 | 1625.49 | 0.56 | 0.46 | 0.66 |
| 123 | Peru | 79757.13 | 7526.55 | 5708.64 | 9975.11 | 1.36 | 1.03 | 1.80 |
| 125 | Colombia | 86507.58 | 2904.33 | 2485.31 | 3405.21 | 0.34 | 0.29 | 0.40 |
| 126 | Costa Rica | 117380.67 | 206.75 | 184.20 | 227.72 | 0.18 | 0.16 | 0.20 |
| 127 | El Salvador | 56443.11 | 397.70 | 312.69 | 515.60 | 0.57 | 0.45 | 0.74 |
| 128 | Guatemala | 65127.76 | 1167.38 | 1019.29 | 1345.94 | 0.63 | 0.55 | 0.72 |
| 129 | Honduras | 34171.60 | 790.76 | 572.85 | 1084.15 | 1.26 | 0.91 | 1.73 |
| 130 | Mexico | 111124.34 | 11046.31 | 9798.72 | 12459.92 | 0.41 | 0.36 | 0.46 |
| 131 | Nicaragua | 36762.82 | 328.63 | 277.96 | 415.53 | 0.70 | 0.59 | 0.88 |
| 132 | Panama | 150795.59 | 857.64 | 701.29 | 1017.52 | 0.65 | 0.53 | 0.77 |
| 133 | Bolivarian Republic of Venezuela | 33286.96 | 1068.98 | 813.35 | 1385.94 | 0.65 | 0.50 | 0.84 |
| 135 | Brazil | 94728.90 | 20947.88 | 19818.46 | 21981.59 | 0.53 | 0.50 | 0.55 |
| 136 | Paraguay | 81293.63 | 798.25 | 614.06 | 1031.58 | 0.72 | 0.56 | 0.93 |
| 139 | Algeria | 74638.48 | 3686.84 | 2772.30 | 5130.19 | 0.58 | 0.43 | 0.80 |
| 140 | Bahrain | 284541.69 | 142.06 | 115.10 | 183.43 | 0.17 | 0.14 | 0.22 |
| 141 | Egypt | 85493.75 | 3390.95 | 2802.86 | 4094.33 | 0.21 | 0.17 | 0.25 |
| 142 | Islamic Republic of Iran | 77037.35 | 2149.00 | 1851.59 | 2589.63 | 0.17 | 0.15 | 0.20 |
| 143 | Iraq | 69514.06 | 2401.28 | 1859.89 | 3124.41 | 0.46 | 0.35 | 0.60 |
| 144 | Jordan | 46355.06 | 87.40 | 70.18 | 111.76 | 0.08 | 0.06 | 0.10 |
| 145 | Kuwait | 242123.59 | 399.46 | 338.39 | 468.66 | 0.17 | 0.14 | 0.20 |
| 146 | Lebanon | 59652.18 | 94.35 | 79.60 | 112.03 | 0.15 | 0.12 | 0.17 |
| 147 | Libya | 68142.83 | 415.36 | 271.34 | 619.02 | 0.48 | 0.31 | 0.71 |
| 148 | Morocco | 45616.42 | 4531.44 | 2970.70 | 8733.32 | 1.41 | 0.93 | 2.72 |
| 149 | Palestine | 29458.97 | 34.41 | 26.32 | 52.02 | 0.12 | 0.09 | 0.18 |
| 150 | Oman | 201100.97 | 160.95 | 126.03 | 223.89 | 0.09 | 0.07 | 0.12 |
| 151 | Qatar | 577980.86 | 643.99 | 472.96 | 866.58 | 0.19 | 0.14 | 0.25 |
| 152 | Saudi Arabia | 290727.34 | 14191.24 | 10467.20 | 21188.36 | 0.67 | 0.50 | 1.01 |
| 153 | Syrian Arab Republic | 24239.67 | 92.79 | 70.10 | 123.29 | 0.14 | 0.11 | 0.19 |
| 154 | Tunisia | 64277.07 | 420.43 | 283.37 | 663.40 | 0.29 | 0.19 | 0.45 |
| 155 | Turkey | 158331.43 | 4669.27 | 3837.22 | 5584.82 | 0.18 | 0.15 | 0.21 |
| 156 | United Arab Emirates | 340462.75 | 575.52 | 304.45 | 894.30 | 0.09 | 0.05 | 0.14 |
| 157 | Yemen | 11143.36 | 430.30 | 284.08 | 652.28 | 0.67 | 0.44 | 1.02 |
| 160 | Afghanistan | 14136.20 | 5974.85 | 4583.65 | 8899.79 | 8.92 | 6.85 | 13.29 |
| 161 | Bangladesh | 39179.49 | 41251.86 | 32914.35 | 51281.49 | 3.37 | 2.69 | 4.19 |
| 162 | Bhutan | 69225.13 | 250.26 | 136.00 | 456.59 | 2.46 | 1.34 | 4.48 |
| 163 | India | 44759.60 | 673981.18 | 586709.43 | 816101.87 | 5.92 | 5.15 | 7.17 |
| 164 | Nepal | 25344.43 | 6044.39 | 4328.11 | 8042.19 | 4.27 | 3.06 | 5.68 |
| 165 | Pakistan | 31389.14 | 92417.81 | 69159.20 | 113698.94 | 7.31 | 5.47 | 8.99 |
| 168 | Angola | 45900.26 | 28296.45 | 19892.01 | 37365.02 | 11.68 | 8.21 | 15.42 |
| 169 | Central African Republic | 8294.91 | 3181.43 | 2127.06 | 4299.74 | 51.40 | 34.37 | 69.47 |
| 170 | Congo | 38253.78 | 3239.82 | 2137.05 | 4765.18 | 9.59 | 6.33 | 14.11 |
| 171 | Democratic Republic of the Congo | 8082.55 | 18279.37 | 12824.52 | 29152.48 | 15.46 | 10.85 | 24.65 |
| 172 | Equatorial Guinea | 103499.12 | 1008.33 | 609.29 | 1674.95 | 3.96 | 2.40 | 6.58 |
| 173 | Gabon | 109994.49 | 1987.36 | 1195.25 | 2944.71 | 5.91 | 3.55 | 8.75 |
| 175 | Burundi | 5047.75 | 1550.28 | 1185.95 | 1991.45 | 14.02 | 10.72 | 18.00 |
| 176 | Comoros | 19422.01 | 228.84 | 165.18 | 308.31 | 8.93 | 6.45 | 12.04 |
| 177 | Djibouti | 34447.01 | 582.55 | 342.07 | 862.97 | 7.78 | 4.57 | 11.53 |
| 178 | Eritrea | 11634.33 | 2475.98 | 1619.14 | 3945.53 | 19.65 | 12.85 | 31.31 |
| 179 | Ethiopia | 15099.06 | 19041.98 | 16435.74 | 22405.46 | 6.75 | 5.83 | 7.95 |
| 180 | Kenya | 31336.33 | 28725.71 | 17599.64 | 37886.93 | 10.75 | 6.58 | 14.18 |
| 181 | Madagascar | 9625.02 | 4802.12 | 3518.24 | 6527.62 | 10.51 | 7.70 | 14.29 |
| 182 | Malawi | 10727.34 | 3230.58 | 2192.29 | 4677.98 | 9.84 | 6.68 | 14.25 |
| 183 | Mauritius | 117503.42 | 73.50 | 65.03 | 81.73 | 0.25 | 0.22 | 0.28 |
| 184 | Mozambique | 9822.77 | 8204.25 | 5864.91 | 10832.19 | 18.12 | 12.95 | 23.92 |
| 185 | Rwanda | 15802.58 | 2355.03 | 1574.22 | 3169.98 | 6.50 | 4.34 | 8.75 |
| 186 | Seychelles | 153409.05 | 23.01 | 19.49 | 27.20 | 0.73 | 0.62 | 0.86 |
| 187 | Somalia | 9672.95 | 9070.04 | 5478.07 | 15886.82 | 30.15 | 18.21 | 52.80 |
| 189 | United Republic of Tanzania | 20686.94 | 13592.70 | 9713.36 | 18178.20 | 6.66 | 4.76 | 8.90 |
| 190 | Uganda | 16509.16 | 9115.85 | 6832.75 | 12521.96 | 7.84 | 5.88 | 10.77 |
| 191 | Zambia | 22575.82 | 3398.61 | 2271.42 | 5181.29 | 4.97 | 3.32 | 7.58 |
| 193 | Botswana | 113585.41 | 4096.65 | 2753.27 | 5611.69 | 9.53 | 6.41 | 13.05 |
| 194 | Lesotho | 19789.98 | 2320.17 | 1565.91 | 2964.87 | 48.64 | 32.83 | 62.16 |
| 195 | Namibia | 60566.22 | 2644.92 | 1801.30 | 3831.60 | 11.21 | 7.63 | 16.23 |
| 196 | South Africa | 86112.84 | 88676.46 | 79443.33 | 101115.29 | 11.38 | 10.19 | 12.97 |
| 197 | Kingdom of Eswatini | 70469.02 | 2213.55 | 1443.87 | 3180.30 | 19.46 | 12.69 | 27.96 |
| 198 | Zimbabwe | 21544.92 | 10974.18 | 7162.38 | 14703.87 | 22.10 | 14.42 | 29.60 |
| 200 | Benin | 20719.39 | 1720.98 | 1209.43 | 2681.11 | 3.68 | 2.59 | 5.73 |
| 201 | Burkina Faso | 15488.73 | 4118.81 | 3004.85 | 5564.43 | 7.28 | 5.31 | 9.83 |
| 202 | Cameroon | 29506.13 | 6770.75 | 4183.21 | 10555.12 | 4.44 | 2.75 | 6.93 |
| 203 | Republic of Cabo Verde | 39613.88 | 76.38 | 52.87 | 129.73 | 1.78 | 1.23 | 3.02 |
| 204 | Chad | 10973.52 | 3577.99 | 2593.36 | 4789.89 | 11.86 | 8.59 | 15.87 |
| 205 | Republic of C涔坱e d'Ivoire | 36202.87 | 8096.94 | 5667.43 | 11855.30 | 4.81 | 3.36 | 7.04 |
| 206 | Republic of the Gambia | 16567.51 | 404.60 | 296.09 | 598.02 | 6.08 | 4.45 | 8.99 |
| 207 | Ghana | 38325.04 | 12274.99 | 9094.44 | 16648.01 | 5.42 | 4.02 | 7.36 |
| 208 | Guinea | 23346.04 | 3161.02 | 2236.03 | 4623.64 | 6.30 | 4.45 | 9.21 |
| 209 | Guinea-Bissau | 15761.35 | 573.53 | 421.10 | 747.55 | 11.34 | 8.32 | 14.78 |
| 210 | Liberia | 9439.81 | 346.90 | 232.40 | 638.34 | 4.13 | 2.77 | 7.60 |
| 211 | Mali | 14949.68 | 3004.92 | 2216.65 | 4279.97 | 5.31 | 3.92 | 7.56 |
| 212 | Mauritania | 31577.61 | 540.88 | 389.63 | 840.28 | 2.11 | 1.52 | 3.27 |
| 213 | Niger | 9604.94 | 3236.92 | 2161.20 | 5380.78 | 8.15 | 5.44 | 13.55 |
| 214 | Nigeria | 33055.51 | 77389.83 | 52210.71 | 102884.75 | 6.10 | 4.11 | 8.10 |
| 215 | Sao Tome and Principe | 30497.41 | 21.49 | 15.28 | 34.92 | 1.73 | 1.23 | 2.81 |
| 216 | Senegal | 23864.84 | 2707.84 | 2030.69 | 3623.03 | 4.09 | 3.07 | 5.47 |
| 217 | Sierra Leone | 17698.33 | 2070.95 | 1508.09 | 3087.60 | 8.20 | 5.97 | 12.22 |
| 218 | Togo | 15333.34 | 1339.88 | 953.71 | 2054.53 | 6.21 | 4.42 | 9.52 |
| 298 | American Samoa | 37286.29 | 1.76 | 1.47 | 2.11 | 0.51 | 0.42 | 0.61 |
| 305 | Bermuda | 439268.63 | 1.93 | 1.32 | 2.59 | 0.03 | 0.02 | 0.04 |
| 349 | Greenland | 353046.66 | 23.29 | 19.67 | 27.93 | 0.61 | 0.52 | 0.73 |
| 351 | Guam | 185099.00 | 60.59 | 52.63 | 69.78 | 1.00 | 0.87 | 1.15 |
| 367 | Principality of Monaco | 1030838.21 | 11.41 | 8.89 | 14.05 | 0.14 | 0.11 | 0.17 |
| 369 | Republic of Nauru | 72789.64 | 7.11 | 4.93 | 9.63 | 5.29 | 3.66 | 7.16 |
| 376 | Northern Mariana Islands | 108563.43 | 16.77 | 14.05 | 20.45 | 1.65 | 1.38 | 2.01 |
| 380 | Republic of Palau | 85593.69 | 5.02 | 4.07 | 6.18 | 1.77 | 1.43 | 2.18 |
| 385 | Puerto Rico | 192605.34 | 93.48 | 78.87 | 108.86 | 0.07 | 0.06 | 0.08 |
| 393 | Saint Kitts and Nevis | 141533.58 | 4.02 | 3.38 | 4.80 | 0.26 | 0.21 | 0.30 |
| 396 | Republic of San Marino | 289996.19 | 0.70 | 0.48 | 0.95 | 0.03 | 0.02 | 0.04 |
| 416 | Tuvalu | 29272.88 | 4.55 | 3.29 | 5.69 | 6.87 | 4.97 | 8.59 |
| 422 | United States Virgin Islands | 233854.30 | 5.47 | 4.11 | 7.18 | 0.14 | 0.10 | 0.18 |
| 435 | South Sudan | 4929.59 | 1388.39 | 973.26 | 2049.11 | 20.05 | 14.05 | 29.59 |
| 522 | Sudan | 18100.95 | 1056.96 | 664.51 | 1555.62 | 0.75 | 0.47 | 1.10 |

Table 2. VLW and VLW/GDP by 204countries or territories 2021 for TB, Generated Using Income Elasticity (IE) of the VSL at 0.55.

| Location_id | Location name | VSLY | VLW region  (millions) | VLW region  (millions)  95%UI lower | VLW region (millions)  95%UI upper | VLW/GDP (%) | VLW/GDP(%) 95%UI lower | VLW/GDP (%) 95%UI upper |
| --- | --- | --- | --- | --- | --- | --- | --- | --- |
| 6 | China | 52187.12 | 71783.91 | 58492.36 | 89922.19 | 0.25 | 0.20 | 0.31 |
| 7 | Democratic People's Republic of Korea | 686.20 | 246.16 | 176.43 | 352.56 | 0.85 | 0.61 | 1.21 |
| 10 | Cambodia | 10044.26 | 2548.50 | 1747.95 | 3676.86 | 2.40 | 1.65 | 3.47 |
| 11 | Indonesia | 28720.71 | 90818.91 | 75769.73 | 108413.45 | 2.55 | 2.13 | 3.05 |
| 12 | Lao People's Democratic Republic | 14836.76 | 1064.52 | 664.59 | 1472.98 | 1.79 | 1.11 | 2.47 |
| 13 | Malaysia | 98752.10 | 6930.42 | 6056.23 | 8162.90 | 0.73 | 0.64 | 0.86 |
| 14 | Maldives | 46559.49 | 36.69 | 29.76 | 45.16 | 0.37 | 0.30 | 0.46 |
| 15 | Myanmar | 7675.59 | 4501.24 | 3462.01 | 6026.64 | 1.54 | 1.18 | 2.06 |
| 16 | Philippines | 17004.57 | 18548.00 | 15792.40 | 21409.82 | 1.85 | 1.57 | 2.13 |
| 17 | Sri Lanka | 31722.95 | 1117.38 | 811.49 | 1537.07 | 0.35 | 0.25 | 0.48 |
| 18 | Thailand | 53135.74 | 13482.24 | 10907.16 | 16800.22 | 1.00 | 0.81 | 1.24 |
| 19 | Timor-Leste | 11506.69 | 270.35 | 197.42 | 379.00 | 2.83 | 2.07 | 3.97 |
| 20 | SoUIalist Republic of Viet Nam | 24742.77 | 15142.16 | 11889.08 | 19885.57 | 1.25 | 0.98 | 1.65 |
| 22 | Fiji | 23289.70 | 44.18 | 34.88 | 56.10 | 0.45 | 0.35 | 0.57 |
| 23 | Kiribati | 3674.61 | 12.88 | 9.75 | 17.37 | 3.49 | 2.64 | 4.70 |
| 24 | Marshall Islands | 11814.61 | 15.79 | 10.38 | 22.02 | 4.18 | 2.75 | 5.84 |
| 25 | Federated States of Micronesia | 5110.34 | 2.61 | 1.96 | 3.46 | 0.65 | 0.49 | 0.86 |
| 26 | Papua New Guinea | 5505.19 | 654.94 | 528.96 | 805.75 | 1.57 | 1.27 | 1.94 |
| 27 | Samoa | 9709.11 | 5.54 | 4.32 | 7.25 | 0.42 | 0.33 | 0.55 |
| 28 | Solomon Islands | 2666.44 | 9.54 | 7.45 | 12.41 | 0.55 | 0.43 | 0.72 |
| 29 | Tonga | 11305.42 | 2.14 | 1.67 | 2.81 | 0.29 | 0.22 | 0.38 |
| 30 | Vanuatu | 3705.64 | 5.45 | 3.51 | 7.57 | 0.56 | 0.36 | 0.77 |
| 33 | Armenia | 37605.71 | 64.42 | 56.42 | 73.25 | 0.14 | 0.12 | 0.15 |
| 34 | Azerbaijan | 56569.65 | 1304.25 | 976.49 | 1871.50 | 0.62 | 0.46 | 0.89 |
| 35 | Georgia | 50977.16 | 229.71 | 203.58 | 257.70 | 0.34 | 0.30 | 0.38 |
| 36 | Kazakhstan | 120306.71 | 3338.38 | 2978.48 | 3694.36 | 0.53 | 0.48 | 0.59 |
| 37 | Kyrgyzstan | 8402.69 | 136.03 | 113.64 | 160.27 | 0.35 | 0.29 | 0.41 |
| 38 | Mongolia | 36035.65 | 778.12 | 550.34 | 1171.06 | 1.58 | 1.12 | 2.37 |
| 39 | Tajikistan | 5052.83 | 198.59 | 148.01 | 259.77 | 0.49 | 0.37 | 0.64 |
| 40 | Turkmenistan | 43152.49 | 820.07 | 658.30 | 1023.13 | 0.97 | 0.78 | 1.21 |
| 41 | Uzbekistan | 17195.69 | 1119.57 | 976.47 | 1275.39 | 0.35 | 0.31 | 0.40 |
| 43 | Albania | 37870.70 | 37.07 | 26.13 | 58.36 | 0.09 | 0.06 | 0.14 |
| 44 | Bosnia and Herzegovina | 46452.88 | 138.99 | 109.65 | 173.00 | 0.23 | 0.18 | 0.29 |
| 45 | Bulgaria | 100454.65 | 279.04 | 243.00 | 319.12 | 0.14 | 0.12 | 0.16 |
| 46 | Croatia | 129932.18 | 182.73 | 158.69 | 206.60 | 0.12 | 0.10 | 0.13 |
| 47 | Czech Republic | 191476.97 | 193.42 | 170.06 | 224.58 | 0.04 | 0.03 | 0.04 |
| 48 | Hungary | 141981.54 | 273.28 | 239.14 | 308.20 | 0.07 | 0.07 | 0.08 |
| 49 | North Macedonia | 64523.41 | 68.01 | 50.57 | 92.33 | 0.14 | 0.10 | 0.19 |
| 50 | Montenegro | 71918.65 | 14.55 | 11.31 | 18.91 | 0.10 | 0.08 | 0.13 |
| 51 | Poland | 155322.52 | 2207.44 | 1989.56 | 2419.67 | 0.14 | 0.13 | 0.15 |
| 52 | Romania | 140740.94 | 4086.46 | 3576.16 | 4565.75 | 0.57 | 0.50 | 0.64 |
| 53 | Serbia | 67716.73 | 227.89 | 180.94 | 281.50 | 0.11 | 0.09 | 0.13 |
| 54 | Slovakia | 141619.30 | 151.13 | 121.63 | 186.53 | 0.07 | 0.06 | 0.09 |
| 55 | Slovenia | 173538.40 | 38.35 | 33.33 | 44.20 | 0.04 | 0.04 | 0.05 |
| 57 | Belarus | 90757.24 | 838.35 | 677.18 | 1019.07 | 0.33 | 0.26 | 0.40 |
| 58 | Estonia | 171659.12 | 117.81 | 103.12 | 133.35 | 0.20 | 0.18 | 0.23 |
| 59 | Latvia | 136493.46 | 218.56 | 193.07 | 242.13 | 0.32 | 0.28 | 0.35 |
| 60 | Lithuania | 188690.40 | 684.88 | 605.03 | 762.49 | 0.55 | 0.48 | 0.61 |
| 61 | Republic of Moldova | 38189.72 | 215.90 | 192.36 | 241.71 | 0.38 | 0.34 | 0.43 |
| 62 | Russian Federation | 154906.43 | 35779.04 | 32913.89 | 38773.12 | 0.63 | 0.58 | 0.69 |
| 63 | Ukraine | 47527.29 | 4581.37 | 3273.72 | 6153.27 | 0.60 | 0.43 | 0.80 |
| 66 | Brunei Darussalam | 401558.89 | 199.60 | 169.19 | 235.76 | 0.57 | 0.48 | 0.67 |
| 67 | Japan | 155062.98 | 8101.57 | 6773.94 | 8932.59 | 0.14 | 0.12 | 0.16 |
| 68 | Republic of Korea | 181492.41 | 9042.99 | 7775.60 | 10584.34 | 0.36 | 0.31 | 0.42 |
| 69 | Singapore | 784024.70 | 1067.09 | 950.54 | 1195.96 | 0.14 | 0.13 | 0.16 |
| 71 | Australia | 243333.22 | 411.76 | 369.10 | 450.79 | 0.03 | 0.02 | 0.03 |
| 72 | New Zealand | 183644.10 | 39.25 | 32.54 | 46.83 | 0.02 | 0.01 | 0.02 |
| 74 | Andorra | 249593.40 | 0.61 | 0.44 | 0.83 | 0.01 | 0.01 | 0.02 |
| 75 | Austria | 276054.11 | 315.64 | 284.96 | 345.19 | 0.06 | 0.05 | 0.06 |
| 76 | Belgium | 262317.15 | 332.29 | 295.50 | 367.79 | 0.05 | 0.04 | 0.05 |
| 77 | Cyprus | 188236.68 | 26.72 | 22.67 | 32.15 | 0.04 | 0.03 | 0.05 |
| 78 | Denmark | 321495.31 | 146.96 | 132.37 | 163.68 | 0.04 | 0.03 | 0.04 |
| 79 | Finland | 236176.19 | 217.29 | 193.27 | 237.95 | 0.07 | 0.06 | 0.08 |
| 80 | France | 209443.08 | 2530.16 | 2230.79 | 2768.19 | 0.07 | 0.06 | 0.08 |
| 81 | Germany | 277106.34 | 2741.91 | 2451.17 | 3030.37 | 0.05 | 0.05 | 0.06 |
| 82 | Greece | 109545.06 | 221.15 | 201.87 | 239.40 | 0.06 | 0.06 | 0.07 |
| 83 | Iceland | 261476.76 | 8.60 | 7.57 | 9.52 | 0.04 | 0.04 | 0.04 |
| 84 | Ireland | 709987.38 | 358.58 | 318.79 | 397.52 | 0.06 | 0.05 | 0.07 |
| 85 | Israel | 169613.18 | 111.06 | 98.05 | 122.16 | 0.03 | 0.02 | 0.03 |
| 86 | Italy | 192618.73 | 1125.26 | 1009.89 | 1220.01 | 0.04 | 0.03 | 0.04 |
| 87 | Luxembourg | 861479.97 | 29.41 | 25.86 | 33.72 | 0.03 | 0.03 | 0.04 |
| 88 | Malta | 232991.85 | 5.33 | 4.63 | 6.22 | 0.02 | 0.02 | 0.02 |
| 89 | Netherlands | 313748.47 | 488.84 | 438.65 | 538.81 | 0.04 | 0.04 | 0.05 |
| 90 | Norway | 456353.30 | 325.06 | 291.24 | 353.31 | 0.07 | 0.06 | 0.07 |
| 91 | Portugal | 133713.70 | 582.73 | 535.03 | 637.88 | 0.14 | 0.13 | 0.16 |
| 92 | Spain | 160575.44 | 957.26 | 860.99 | 1051.93 | 0.05 | 0.04 | 0.05 |
| 93 | Sweden | 269079.34 | 248.40 | 214.66 | 279.96 | 0.04 | 0.03 | 0.04 |
| 94 | Switzerland | 392113.15 | 249.06 | 220.72 | 277.72 | 0.03 | 0.03 | 0.04 |
| 95 | United Kingdom of Great Britain and Northern Ireland | 205463.52 | 1443.08 | 1343.41 | 1546.98 | 0.04 | 0.04 | 0.04 |
| 97 | Argentina | 79452.09 | 2095.85 | 1952.76 | 2248.53 | 0.18 | 0.16 | 0.19 |
| 98 | Chile | 90148.36 | 1147.19 | 1059.01 | 1243.40 | 0.21 | 0.19 | 0.23 |
| 99 | Uruguay | 94622.94 | 163.97 | 151.66 | 178.34 | 0.16 | 0.15 | 0.18 |
| 101 | Canada | 236162.20 | 626.57 | 569.73 | 688.08 | 0.03 | 0.03 | 0.03 |
| 102 | United States of America | 362772.14 | 8239.95 | 7628.12 | 8831.68 | 0.03 | 0.03 | 0.04 |
| 105 | Antigua and Barbuda | 77824.50 | 1.16 | 0.99 | 1.38 | 0.05 | 0.04 | 0.06 |
| 106 | Commonwealth of the Bahamas | 101038.99 | 24.01 | 19.54 | 29.75 | 0.21 | 0.17 | 0.26 |
| 107 | Barbados | 36501.41 | 1.98 | 1.60 | 2.45 | 0.04 | 0.03 | 0.05 |
| 108 | Belize | 24331.99 | 12.12 | 10.72 | 13.54 | 0.24 | 0.21 | 0.27 |
| 109 | Cuba | 5301.45 | 6.81 | 5.81 | 8.02 | 0.01 | 0.01 | 0.02 |
| 110 | Dominica | 39039.15 | 3.14 | 2.42 | 4.14 | 0.30 | 0.23 | 0.40 |
| 111 | Dominican Republic | 62298.93 | 1788.18 | 1310.78 | 2775.57 | 0.74 | 0.54 | 1.15 |
| 112 | Grenada | 38023.47 | 0.69 | 0.57 | 0.80 | 0.04 | 0.04 | 0.05 |
| 113 | Guyana | 76133.57 | 159.02 | 123.27 | 203.39 | 0.91 | 0.70 | 1.16 |
| 114 | Haiti | 4138.95 | 325.01 | 197.36 | 916.85 | 0.81 | 0.49 | 2.27 |
| 115 | Jamaica | 17780.03 | 8.93 | 7.05 | 11.19 | 0.03 | 0.03 | 0.04 |
| 116 | Saint Lucia | 51376.65 | 6.77 | 5.66 | 8.06 | 0.20 | 0.17 | 0.24 |
| 117 | Saint Vincent and the Grenadines | 39264.84 | 2.61 | 2.31 | 2.97 | 0.14 | 0.13 | 0.16 |
| 118 | Suriname | 50509.35 | 20.39 | 15.64 | 25.62 | 0.19 | 0.15 | 0.24 |
| 119 | Trinidad and Tobago | 108052.43 | 66.49 | 52.18 | 83.92 | 0.15 | 0.12 | 0.20 |
| 121 | Plurinational State of Bolivia | 19463.19 | 1101.40 | 769.10 | 1497.40 | 0.99 | 0.69 | 1.34 |
| 122 | Ecuador | 30061.18 | 590.99 | 488.86 | 706.01 | 0.24 | 0.20 | 0.29 |
| 123 | Peru | 36802.92 | 3473.03 | 2634.18 | 4602.89 | 0.63 | 0.48 | 0.83 |
| 125 | Colombia | 42573.21 | 1429.31 | 1223.10 | 1675.82 | 0.17 | 0.14 | 0.20 |
| 126 | Costa Rica | 67668.43 | 119.19 | 106.19 | 131.28 | 0.11 | 0.09 | 0.12 |
| 127 | El Salvador | 21904.67 | 154.34 | 121.35 | 200.10 | 0.22 | 0.17 | 0.29 |
| 128 | Guatemala | 26439.10 | 473.90 | 413.79 | 546.40 | 0.25 | 0.22 | 0.29 |
| 129 | Honduras | 10045.79 | 232.47 | 168.41 | 318.72 | 0.37 | 0.27 | 0.51 |
| 130 | Mexico | 59796.77 | 5944.10 | 5272.76 | 6704.77 | 0.22 | 0.20 | 0.25 |
| 131 | Nicaragua | 11551.73 | 103.26 | 87.34 | 130.57 | 0.22 | 0.18 | 0.28 |
| 132 | Panama | 98995.79 | 563.03 | 460.39 | 667.99 | 0.42 | 0.35 | 0.50 |
| 133 | Bolivarian Republic of Venezuela | 9755.72 | 313.30 | 238.37 | 406.19 | 0.19 | 0.15 | 0.25 |
| 135 | Brazil | 47538.87 | 10512.51 | 9945.72 | 11031.27 | 0.26 | 0.25 | 0.28 |
| 136 | Paraguay | 37663.66 | 369.83 | 284.49 | 477.94 | 0.33 | 0.26 | 0.43 |
| 139 | Algeria | 33544.29 | 1656.95 | 1245.94 | 2305.63 | 0.26 | 0.19 | 0.36 |
| 140 | Bahrain | 248981.43 | 124.31 | 100.71 | 160.50 | 0.15 | 0.12 | 0.19 |
| 141 | Egypt | 39831.18 | 1579.83 | 1305.84 | 1907.53 | 0.10 | 0.08 | 0.12 |
| 142 | Islamic Republic of Iran | 35161.57 | 980.85 | 845.11 | 1181.97 | 0.08 | 0.07 | 0.09 |
| 143 | Iraq | 29277.60 | 1011.36 | 783.34 | 1315.93 | 0.19 | 0.15 | 0.25 |
| 144 | Jordan | 16580.45 | 31.26 | 25.10 | 39.98 | 0.03 | 0.02 | 0.04 |
| 145 | Kuwait | 203475.86 | 335.70 | 284.38 | 393.85 | 0.14 | 0.12 | 0.17 |
| 146 | Lebanon | 23981.14 | 37.93 | 32.00 | 45.04 | 0.06 | 0.05 | 0.07 |
| 147 | Libya | 28598.18 | 174.32 | 113.88 | 259.79 | 0.20 | 0.13 | 0.30 |
| 148 | Morocco | 15811.60 | 1570.69 | 1029.71 | 3027.15 | 0.49 | 0.32 | 0.94 |
| 149 | Palestine | 8274.93 | 9.67 | 7.39 | 14.61 | 0.03 | 0.03 | 0.05 |
| 150 | Oman | 147705.79 | 118.22 | 92.57 | 164.45 | 0.06 | 0.05 | 0.09 |
| 151 | Qatar | 737418.71 | 821.63 | 603.43 | 1105.63 | 0.24 | 0.17 | 0.32 |
| 152 | Saudi Arabia | 256270.17 | 12509.29 | 9226.62 | 18677.10 | 0.59 | 0.44 | 0.89 |
| 153 | Syrian Arab Republic | 6131.77 | 23.47 | 17.73 | 31.19 | 0.04 | 0.03 | 0.05 |
| 154 | Tunisia | 26690.30 | 174.58 | 117.67 | 275.47 | 0.12 | 0.08 | 0.19 |
| 155 | Turkey | 104622.08 | 3085.35 | 2535.56 | 3690.33 | 0.12 | 0.10 | 0.14 |
| 156 | United Arab Emirates | 329930.06 | 557.71 | 295.03 | 866.63 | 0.09 | 0.05 | 0.13 |
| 157 | Yemen | 1814.49 | 70.07 | 46.26 | 106.21 | 0.11 | 0.07 | 0.17 |
| 160 | Afghanistan | 2443.32 | 1032.70 | 792.25 | 1538.25 | 1.54 | 1.18 | 2.30 |
| 161 | Bangladesh | 12615.23 | 13282.50 | 10597.95 | 16511.90 | 1.08 | 0.87 | 1.35 |
| 162 | Bhutan | 29976.84 | 108.37 | 58.89 | 197.72 | 1.06 | 0.58 | 1.94 |
| 163 | India | 14990.06 | 225717.35 | 196489.91 | 273313.79 | 1.98 | 1.73 | 2.40 |
| 164 | Nepal | 6378.62 | 1521.24 | 1089.29 | 2024.04 | 1.07 | 0.77 | 1.43 |
| 165 | Pakistan | 8583.71 | 25272.68 | 18912.35 | 31092.24 | 2.00 | 1.50 | 2.46 |
| 168 | Angola | 14746.47 | 9090.86 | 6390.75 | 12004.34 | 3.75 | 2.64 | 4.95 |
| 169 | Central African Republic | 1040.14 | 398.93 | 266.72 | 539.17 | 6.45 | 4.31 | 8.71 |
| 170 | Congo | 11300.34 | 957.06 | 631.29 | 1407.66 | 2.83 | 1.87 | 4.17 |
| 171 | Democratic Republic of the Congo | 1093.46 | 2472.95 | 1734.98 | 3943.93 | 2.09 | 1.47 | 3.34 |
| 172 | Equatorial Guinea | 50105.69 | 488.15 | 294.97 | 810.87 | 1.92 | 1.16 | 3.19 |
| 173 | Gabon | 55879.45 | 1009.62 | 607.21 | 1495.97 | 3.00 | 1.81 | 4.45 |
| 175 | Burundi | 544.99 | 167.38 | 128.04 | 215.01 | 1.51 | 1.16 | 1.94 |
| 176 | Comoros | 4252.74 | 50.11 | 36.17 | 67.51 | 1.96 | 1.41 | 2.64 |
| 177 | Djibouti | 9916.32 | 167.70 | 98.47 | 248.42 | 2.24 | 1.32 | 3.32 |
| 178 | Eritrea | 1897.92 | 403.91 | 264.13 | 643.64 | 3.21 | 2.10 | 5.11 |
| 179 | Ethiopia | 2867.38 | 3616.16 | 3121.22 | 4254.90 | 1.28 | 1.11 | 1.51 |
| 180 | Kenya | 8546.55 | 7834.54 | 4800.06 | 10333.13 | 2.93 | 1.80 | 3.87 |
| 181 | Madagascar | 1436.81 | 716.86 | 525.20 | 974.44 | 1.57 | 1.15 | 2.13 |
| 182 | Malawi | 1644.92 | 495.37 | 336.16 | 717.32 | 1.51 | 1.02 | 2.19 |
| 183 | Mauritius | 66136.51 | 41.37 | 36.60 | 46.00 | 0.14 | 0.13 | 0.16 |
| 184 | Mozambique | 1399.64 | 1169.02 | 835.69 | 1543.48 | 2.58 | 1.85 | 3.41 |
| 185 | Rwanda | 3082.58 | 459.39 | 307.08 | 618.36 | 1.27 | 0.85 | 1.71 |
| 186 | Seychelles | 99148.07 | 14.87 | 12.59 | 17.58 | 0.47 | 0.40 | 0.56 |
| 187 | Somalia | 1347.32 | 1263.34 | 763.02 | 2212.83 | 4.20 | 2.54 | 7.35 |
| 189 | United Republic of Tanzania | 4563.73 | 2998.67 | 2142.85 | 4010.27 | 1.47 | 1.05 | 1.96 |
| 190 | Uganda | 3193.08 | 1763.12 | 1321.54 | 2421.90 | 1.52 | 1.14 | 2.08 |
| 191 | Zambia | 4987.52 | 750.83 | 501.81 | 1144.67 | 1.10 | 0.73 | 1.67 |
| 193 | Botswana | 56821.26 | 2049.35 | 1377.33 | 2807.26 | 4.77 | 3.20 | 6.53 |
| 194 | Lesotho | 3726.40 | 436.88 | 294.86 | 558.28 | 9.16 | 6.18 | 11.70 |
| 195 | Namibia | 22276.08 | 972.79 | 662.51 | 1409.25 | 4.12 | 2.81 | 5.97 |
| 196 | South Africa | 37637.94 | 38758.44 | 34722.85 | 44195.17 | 4.97 | 4.45 | 5.67 |
| 197 | Kingdom of Eswatini | 26100.66 | 819.87 | 534.79 | 1177.94 | 7.21 | 4.70 | 10.36 |
| 198 | Zimbabwe | 4538.41 | 2311.70 | 1508.75 | 3097.35 | 4.65 | 3.04 | 6.24 |
| 200 | Benin | 4552.00 | 378.09 | 265.71 | 589.03 | 0.81 | 0.57 | 1.26 |
| 201 | Burkina Faso | 2882.85 | 766.62 | 559.28 | 1035.68 | 1.35 | 0.99 | 1.83 |
| 202 | Cameroon | 7625.35 | 1749.78 | 1081.08 | 2727.79 | 1.15 | 0.71 | 1.79 |
| 203 | Republic of Cabo Verde | 12962.83 | 24.99 | 17.30 | 42.45 | 0.58 | 0.40 | 0.99 |
| 204 | Chad | 1688.93 | 550.69 | 399.14 | 737.21 | 1.82 | 1.32 | 2.44 |
| 205 | Republic of C涔坱e d'Ivoire | 10506.72 | 2349.88 | 1644.79 | 3440.62 | 1.40 | 0.98 | 2.04 |
| 206 | Republic of the Gambia | 3259.31 | 79.60 | 58.25 | 117.65 | 1.20 | 0.88 | 1.77 |
| 207 | Ghana | 11628.40 | 3724.42 | 2759.39 | 5051.26 | 1.65 | 1.22 | 2.23 |
| 208 | Guinea | 5328.42 | 721.46 | 510.34 | 1055.28 | 1.44 | 1.02 | 2.10 |
| 209 | Guinea-Bissau | 2912.61 | 105.98 | 77.82 | 138.14 | 2.10 | 1.54 | 2.73 |
| 210 | Liberia | 1382.39 | 50.80 | 34.03 | 93.48 | 0.60 | 0.40 | 1.11 |
| 211 | Mali | 2703.74 | 543.46 | 400.90 | 774.06 | 0.96 | 0.71 | 1.37 |
| 212 | Mauritania | 9004.85 | 154.24 | 111.11 | 239.62 | 0.60 | 0.43 | 0.93 |
| 213 | Niger | 1427.61 | 481.11 | 321.23 | 799.76 | 1.21 | 0.81 | 2.01 |
| 214 | Nigeria | 9143.57 | 21407.01 | 14442.15 | 28459.23 | 1.69 | 1.14 | 2.24 |
| 215 | Sao Tome and Principe | 8619.08 | 6.07 | 4.32 | 9.87 | 0.49 | 0.35 | 0.79 |
| 216 | Senegal | 5754.89 | 652.98 | 489.69 | 873.67 | 0.99 | 0.74 | 1.32 |
| 217 | Sierra Leone | 3525.94 | 412.58 | 300.45 | 615.13 | 1.63 | 1.19 | 2.44 |
| 218 | Togo | 2905.56 | 253.90 | 180.72 | 389.32 | 1.18 | 0.84 | 1.80 |
| 298 | American Samoa | 11642.73 | 0.55 | 0.46 | 0.66 | 0.16 | 0.13 | 0.19 |
| 305 | Bermuda | 498073.98 | 2.18 | 1.50 | 2.93 | 0.04 | 0.03 | 0.05 |
| 349 | Greenland | 343859.65 | 22.68 | 19.16 | 27.20 | 0.59 | 0.50 | 0.71 |
| 351 | Guam | 134816.41 | 44.13 | 38.34 | 50.82 | 0.73 | 0.63 | 0.84 |
| 367 | Principality of Monaco | 1779848.86 | 19.71 | 15.36 | 24.27 | 0.24 | 0.19 | 0.30 |
| 369 | Republic of Nauru | 30006.03 | 2.93 | 2.03 | 3.97 | 2.18 | 1.51 | 2.95 |
| 376 | Northern Mariana Islands | 58647.95 | 9.06 | 7.59 | 11.05 | 0.89 | 0.75 | 1.09 |
| 380 | Republic of Palau | 39985.30 | 2.35 | 1.90 | 2.89 | 0.83 | 0.67 | 1.02 |
| 385 | Puerto Rico | 144788.61 | 70.27 | 59.29 | 81.83 | 0.05 | 0.04 | 0.06 |
| 393 | Saint Kitts and Nevis | 86641.72 | 2.46 | 2.07 | 2.94 | 0.16 | 0.13 | 0.19 |
| 396 | Republic of San Marino | 275432.12 | 0.67 | 0.46 | 0.90 | 0.03 | 0.02 | 0.04 |
| 416 | Tuvalu | 7994.32 | 1.24 | 0.90 | 1.55 | 1.88 | 1.36 | 2.35 |
| 422 | United States Virgin Islands | 189025.61 | 4.42 | 3.32 | 5.80 | 0.11 | 0.08 | 0.14 |
| 435 | South Sudan | 492.36 | 138.67 | 97.21 | 204.66 | 2.00 | 1.40 | 2.96 |
| 522 | Sudan | 3851.18 | 224.88 | 141.38 | 330.97 | 0.16 | 0.10 | 0.23 |

Table 3. VLW and VLW/GDP by 204countries or territories 2021 for TB, Generated Using Income Elasticity (IE) of the VSL at 1.50.

| Location_id | Location name | VSLY | VLW region  (millions) | VLW region  (millions)  95%UI lower | VLW region (millions)  95%UI upper | VLW/GDP (%) | VLW/GDP(%) 95%UI lower | VLW/GDP (%) 95%UI upper |
| --- | --- | --- | --- | --- | --- | --- | --- | --- |
| 6 | China | 172362.59 | 237086.49 | 193187.40 | 296993.21 | 0.82 | 0.67 | 1.02 |
| 7 | Democratic People's Republic of Korea | 36332.45 | 13033.35 | 9341.30 | 18667.15 | 44.89 | 32.18 | 64.30 |
| 10 | Cambodia | 102474.19 | 26000.51 | 17833.08 | 37512.29 | 24.50 | 16.81 | 35.35 |
| 11 | Indonesia | 148216.33 | 468680.78 | 391017.87 | 559479.32 | 13.17 | 10.99 | 15.72 |
| 12 | Lao People's Democratic Republic | 118151.81 | 8477.29 | 5292.43 | 11730.01 | 14.22 | 8.88 | 19.68 |
| 13 | Malaysia | 227451.63 | 15962.56 | 13949.06 | 18801.27 | 1.68 | 1.47 | 1.98 |
| 14 | Maldives | 164140.43 | 129.33 | 104.91 | 159.21 | 1.31 | 1.06 | 1.62 |
| 15 | Myanmar | 93279.54 | 54702.53 | 42072.99 | 73240.28 | 18.72 | 14.40 | 25.07 |
| 16 | Philippines | 124099.79 | 135363.78 | 115253.28 | 156249.42 | 13.49 | 11.49 | 15.58 |
| 17 | Sri Lanka | 146724.78 | 5168.08 | 3753.30 | 7109.22 | 1.62 | 1.18 | 2.23 |
| 18 | Thailand | 176825.27 | 44866.24 | 36296.87 | 55907.82 | 3.32 | 2.69 | 4.14 |
| 19 | Timor-Leste | 107579.62 | 2527.58 | 1845.76 | 3543.42 | 26.50 | 19.35 | 37.15 |
| 20 | Socialist Republic of Viet Nam | 134807.07 | 82499.64 | 64775.78 | 108343.35 | 6.83 | 5.36 | 8.97 |
| 22 | Fiji | 142227.94 | 269.81 | 213.01 | 342.60 | 2.73 | 2.16 | 3.47 |
| 23 | Kiribati | 73851.96 | 258.85 | 195.89 | 349.15 | 70.06 | 53.02 | 94.50 |
| 24 | Marshall Islands | 112318.43 | 150.11 | 98.65 | 209.38 | 39.78 | 26.14 | 55.49 |
| 25 | Federated States of Micronesia | 80874.39 | 41.38 | 31.07 | 54.77 | 10.29 | 7.72 | 13.62 |
| 26 | Papua New Guinea | 85974.28 | 10228.10 | 8260.81 | 12583.36 | 24.58 | 19.86 | 30.25 |
| 27 | Samoa | 99229.29 | 56.65 | 44.18 | 74.07 | 4.27 | 3.33 | 5.58 |
| 28 | Solomon Islands | 64272.47 | 229.84 | 179.65 | 299.15 | 13.35 | 10.44 | 17.38 |
| 29 | Tonga | 102696.28 | 19.44 | 15.16 | 25.52 | 2.60 | 2.03 | 3.41 |
| 30 | Vanuatu | 72525.67 | 106.66 | 68.63 | 148.23 | 10.87 | 6.99 | 15.10 |
| 33 | Armenia | 157227.34 | 269.34 | 235.89 | 306.26 | 0.56 | 0.49 | 0.64 |
| 34 | Azerbaijan | 189442.86 | 4367.73 | 3270.10 | 6267.35 | 2.07 | 1.55 | 2.97 |
| 35 | Georgia | 180950.47 | 815.37 | 722.62 | 914.74 | 1.19 | 1.06 | 1.34 |
| 36 | Kazakhstan | 252082.99 | 6995.03 | 6240.92 | 7740.93 | 1.12 | 1.00 | 1.24 |
| 37 | Kyrgyzstan | 92655.16 | 1500.01 | 1253.04 | 1767.22 | 3.81 | 3.18 | 4.49 |
| 38 | Mongolia | 161571.28 | 3488.81 | 2467.51 | 5250.61 | 7.07 | 5.00 | 10.64 |
| 39 | Tajikistan | 78740.36 | 3094.78 | 2306.57 | 4048.10 | 7.64 | 5.70 | 10.00 |
| 40 | Turkmenistan | 175069.56 | 3327.02 | 2670.71 | 4150.84 | 3.92 | 3.15 | 4.90 |
| 41 | Uzbekistan | 120463.23 | 7843.04 | 6840.61 | 8934.63 | 2.48 | 2.16 | 2.82 |
| 43 | Albania | 156412.90 | 153.10 | 107.93 | 241.02 | 0.36 | 0.25 | 0.56 |
| 44 | Bosnia and Herzegovina | 170272.22 | 509.46 | 401.91 | 634.12 | 0.84 | 0.67 | 1.05 |
| 45 | Bulgaria | 235252.81 | 653.47 | 569.07 | 747.34 | 0.33 | 0.29 | 0.38 |
| 46 | Croatia | 244253.60 | 343.50 | 298.32 | 388.38 | 0.22 | 0.19 | 0.25 |
| 47 | Czech Republic | 281746.61 | 284.60 | 250.24 | 330.45 | 0.06 | 0.05 | 0.07 |
| 48 | Hungary | 257689.70 | 495.99 | 434.03 | 559.37 | 0.13 | 0.12 | 0.15 |
| 49 | North Macedonia | 197188.98 | 207.86 | 154.54 | 282.16 | 0.43 | 0.32 | 0.59 |
| 50 | Montenegro | 203062.85 | 41.08 | 31.93 | 53.38 | 0.28 | 0.21 | 0.36 |
| 51 | Poland | 264031.05 | 3752.40 | 3382.02 | 4113.17 | 0.24 | 0.22 | 0.26 |
| 52 | Romania | 259241.45 | 7527.17 | 6587.19 | 8410.01 | 1.05 | 0.92 | 1.18 |
| 53 | Serbia | 196332.52 | 660.73 | 524.60 | 816.15 | 0.32 | 0.25 | 0.39 |
| 54 | Slovakia | 256829.36 | 274.08 | 220.58 | 338.27 | 0.13 | 0.11 | 0.16 |
| 55 | Slovenia | 265284.34 | 58.63 | 50.96 | 67.56 | 0.06 | 0.05 | 0.07 |
| 57 | Belarus | 224913.22 | 2077.59 | 1678.18 | 2525.44 | 0.81 | 0.65 | 0.98 |
| 58 | Estonia | 271768.63 | 186.52 | 163.25 | 211.12 | 0.32 | 0.28 | 0.36 |
| 59 | Latvia | 256712.77 | 411.06 | 363.12 | 455.39 | 0.60 | 0.53 | 0.66 |
| 60 | Lithuania | 287952.49 | 1045.17 | 923.31 | 1163.61 | 0.83 | 0.74 | 0.93 |
| 61 | Republic of Moldova | 161986.06 | 915.74 | 815.91 | 1025.24 | 1.63 | 1.45 | 1.82 |
| 62 | Russian Federation | 276932.32 | 63963.60 | 58841.45 | 69316.23 | 1.13 | 1.04 | 1.23 |
| 63 | Ukraine | 178292.94 | 17186.48 | 12280.99 | 23083.26 | 2.24 | 1.60 | 3.00 |
| 66 | Brunei Darussalam | 369921.75 | 183.87 | 155.86 | 217.18 | 0.52 | 0.44 | 0.62 |
| 67 | Japan | 245157.21 | 12808.73 | 10709.71 | 14122.58 | 0.23 | 0.19 | 0.25 |
| 68 | Republic of Korea | 263786.38 | 13143.34 | 11301.28 | 15383.60 | 0.53 | 0.45 | 0.62 |
| 69 | Singapore | 439920.22 | 598.75 | 533.35 | 671.06 | 0.08 | 0.07 | 0.09 |
| 71 | Australia | 297039.50 | 502.64 | 450.57 | 550.29 | 0.03 | 0.03 | 0.04 |
| 72 | New Zealand | 269484.29 | 57.60 | 47.75 | 68.72 | 0.02 | 0.02 | 0.03 |
| 74 | Andorra | 299069.10 | 0.74 | 0.52 | 0.99 | 0.01 | 0.01 | 0.02 |
| 75 | Austria | 312599.69 | 357.42 | 322.68 | 390.88 | 0.06 | 0.06 | 0.07 |
| 76 | Belgium | 307744.79 | 389.83 | 346.68 | 431.49 | 0.06 | 0.05 | 0.06 |
| 77 | Cyprus | 271644.07 | 38.56 | 32.72 | 46.39 | 0.06 | 0.05 | 0.07 |
| 78 | Denmark | 330505.02 | 151.08 | 136.08 | 168.27 | 0.04 | 0.03 | 0.04 |
| 79 | Finland | 295011.88 | 271.42 | 241.42 | 297.22 | 0.09 | 0.08 | 0.09 |
| 80 | France | 281801.22 | 3404.28 | 3001.48 | 3724.54 | 0.10 | 0.09 | 0.11 |
| 81 | Germany | 315879.76 | 3125.56 | 2794.14 | 3454.39 | 0.06 | 0.05 | 0.06 |
| 82 | Greece | 225725.76 | 455.69 | 415.97 | 493.29 | 0.13 | 0.12 | 0.14 |
| 83 | Iceland | 301681.48 | 9.92 | 8.74 | 10.99 | 0.05 | 0.04 | 0.05 |
| 84 | Ireland | 440230.82 | 222.34 | 197.67 | 246.49 | 0.04 | 0.03 | 0.04 |
| 85 | Israel | 258032.00 | 168.96 | 149.16 | 185.84 | 0.04 | 0.03 | 0.04 |
| 86 | Italy | 272448.68 | 1591.61 | 1428.43 | 1725.64 | 0.05 | 0.05 | 0.06 |
| 87 | Luxembourg | 471044.87 | 16.08 | 14.14 | 18.44 | 0.02 | 0.02 | 0.02 |
| 88 | Malta | 291237.17 | 6.66 | 5.78 | 7.77 | 0.03 | 0.02 | 0.03 |
| 89 | Netherlands | 327640.16 | 510.49 | 458.08 | 562.66 | 0.04 | 0.04 | 0.05 |
| 90 | Norway | 372064.84 | 265.02 | 237.45 | 288.05 | 0.05 | 0.05 | 0.06 |
| 91 | Portugal | 240694.66 | 1048.97 | 963.09 | 1148.23 | 0.26 | 0.23 | 0.28 |
| 92 | Spain | 254077.72 | 1514.66 | 1362.35 | 1664.46 | 0.08 | 0.07 | 0.08 |
| 93 | Sweden | 305796.18 | 282.30 | 243.96 | 318.17 | 0.04 | 0.04 | 0.05 |
| 94 | Switzerland | 350324.68 | 222.51 | 197.20 | 248.12 | 0.03 | 0.03 | 0.03 |
| 95 | United Kingdom of Great Britain and Northern Ireland | 284401.61 | 1997.51 | 1859.54 | 2141.32 | 0.06 | 0.05 | 0.06 |
| 97 | Argentina | 206208.94 | 5439.55 | 5068.18 | 5835.80 | 0.45 | 0.42 | 0.49 |
| 98 | Chile | 212480.95 | 2703.94 | 2496.11 | 2930.71 | 0.49 | 0.46 | 0.54 |
| 99 | Uruguay | 220622.21 | 382.30 | 353.61 | 415.81 | 0.38 | 0.35 | 0.41 |
| 101 | Canada | 296061.09 | 785.49 | 714.24 | 862.60 | 0.04 | 0.03 | 0.04 |
| 102 | United States of America | 364970.16 | 8289.88 | 7674.34 | 8885.19 | 0.03 | 0.03 | 0.04 |
| 105 | Antigua and Barbuda | 206210.19 | 3.07 | 2.62 | 3.64 | 0.13 | 0.11 | 0.16 |
| 106 | Commonwealth of the Bahamas | 236352.20 | 56.17 | 45.70 | 69.59 | 0.49 | 0.40 | 0.61 |
| 107 | Barbados | 154807.87 | 8.42 | 6.80 | 10.41 | 0.18 | 0.15 | 0.22 |
| 108 | Belize | 136571.80 | 68.03 | 60.18 | 75.99 | 1.36 | 1.20 | 1.52 |
| 109 | Cuba | 77246.77 | 99.21 | 84.67 | 116.88 | 0.21 | 0.18 | 0.24 |
| 110 | Dominica | 166474.42 | 13.40 | 10.30 | 17.64 | 1.28 | 0.98 | 1.69 |
| 111 | Dominican Republic | 192305.73 | 5519.79 | 4046.13 | 8567.70 | 2.29 | 1.68 | 3.55 |
| 112 | Grenada | 165123.90 | 2.98 | 2.49 | 3.48 | 0.19 | 0.16 | 0.22 |
| 113 | Guyana | 225689.38 | 471.40 | 365.42 | 602.91 | 2.70 | 2.09 | 3.45 |
| 114 | Haiti | 81028.03 | 6362.70 | 3863.65 | 17949.10 | 15.78 | 9.58 | 44.51 |
| 115 | Jamaica | 120481.88 | 60.48 | 47.76 | 75.80 | 0.23 | 0.18 | 0.28 |
| 116 | Saint Lucia | 180685.49 | 23.82 | 19.92 | 28.36 | 0.70 | 0.59 | 0.84 |
| 117 | Saint Vincent and the Grenadines | 164367.37 | 10.94 | 9.69 | 12.44 | 0.60 | 0.53 | 0.69 |
| 118 | Suriname | 183508.21 | 74.08 | 56.83 | 93.07 | 0.69 | 0.53 | 0.87 |
| 119 | Trinidad and Tobago | 241170.85 | 148.40 | 116.47 | 187.30 | 0.35 | 0.27 | 0.44 |
| 121 | Plurinational State of Bolivia | 133516.51 | 7555.57 | 5276.02 | 10272.09 | 6.77 | 4.73 | 9.21 |
| 122 | Ecuador | 146602.56 | 2882.13 | 2384.07 | 3443.05 | 1.18 | 0.97 | 1.41 |
| 123 | Peru | 159980.88 | 15097.13 | 11450.68 | 20008.59 | 2.72 | 2.07 | 3.61 |
| 125 | Colombia | 163749.56 | 5497.58 | 4704.43 | 6445.70 | 0.64 | 0.55 | 0.76 |
| 126 | Costa Rica | 192701.89 | 339.42 | 302.40 | 373.84 | 0.30 | 0.27 | 0.33 |
| 127 | El Salvador | 132305.45 | 932.23 | 732.96 | 1208.59 | 1.34 | 1.05 | 1.73 |
| 128 | Guatemala | 146599.93 | 2627.72 | 2294.38 | 3029.66 | 1.41 | 1.23 | 1.62 |
| 129 | Honduras | 102843.84 | 2379.89 | 1724.07 | 3262.90 | 3.79 | 2.75 | 5.20 |
| 130 | Mexico | 194100.87 | 19294.59 | 17115.43 | 21763.74 | 0.72 | 0.64 | 0.81 |
| 131 | Nicaragua | 104206.37 | 931.51 | 787.89 | 1177.86 | 1.97 | 1.67 | 2.49 |
| 132 | Panama | 220233.50 | 1252.56 | 1024.21 | 1486.06 | 0.94 | 0.77 | 1.12 |
| 133 | Bolivarian Republic of Venezuela | 100458.65 | 3226.14 | 2454.64 | 4182.71 | 1.97 | 1.50 | 2.55 |
| 135 | Brazil | 176186.55 | 38961.01 | 36860.42 | 40883.63 | 0.98 | 0.93 | 1.03 |
| 136 | Paraguay | 162471.46 | 1595.36 | 1227.24 | 2061.70 | 1.44 | 1.11 | 1.87 |
| 139 | Algeria | 153310.74 | 7572.93 | 5694.42 | 10537.64 | 1.18 | 0.89 | 1.64 |
| 140 | Bahrain | 320868.40 | 160.20 | 129.79 | 206.84 | 0.19 | 0.15 | 0.25 |
| 141 | Egypt | 170010.02 | 6743.14 | 5573.67 | 8141.85 | 0.41 | 0.34 | 0.49 |
| 142 | Islamic Republic of Iran | 156052.66 | 4353.17 | 3750.73 | 5245.76 | 0.34 | 0.29 | 0.41 |
| 143 | Iraq | 151375.67 | 5229.09 | 4050.15 | 6803.81 | 1.00 | 0.77 | 1.30 |
| 144 | Jordan | 116935.91 | 220.49 | 177.05 | 281.94 | 0.19 | 0.16 | 0.25 |
| 145 | Kuwait | 283145.00 | 467.14 | 395.72 | 548.06 | 0.20 | 0.17 | 0.23 |
| 146 | Lebanon | 135458.78 | 214.24 | 180.75 | 254.39 | 0.33 | 0.28 | 0.40 |
| 147 | Libya | 148865.40 | 907.39 | 592.77 | 1352.32 | 1.04 | 0.68 | 1.56 |
| 148 | Morocco | 118372.84 | 11758.90 | 7708.85 | 22662.62 | 3.67 | 2.40 | 7.07 |
| 149 | Palestine | 92368.85 | 107.89 | 82.53 | 163.10 | 0.37 | 0.28 | 0.56 |
| 150 | Oman | 265478.36 | 212.47 | 166.37 | 295.57 | 0.12 | 0.09 | 0.16 |
| 151 | Qatar | 464186.84 | 517.20 | 379.84 | 695.97 | 0.15 | 0.11 | 0.20 |
| 152 | Saudi Arabia | 325682.86 | 15897.52 | 11725.72 | 23735.94 | 0.76 | 0.56 | 1.13 |
| 153 | Syrian Arab Republic | 83516.68 | 319.72 | 241.52 | 424.79 | 0.50 | 0.37 | 0.66 |
| 154 | Tunisia | 141771.34 | 927.31 | 625.01 | 1463.22 | 0.63 | 0.43 | 1.00 |
| 155 | Turkey | 229888.13 | 6779.51 | 5571.42 | 8108.84 | 0.26 | 0.21 | 0.31 |
| 156 | United Arab Emirates | 350229.34 | 592.03 | 313.18 | 919.95 | 0.09 | 0.05 | 0.14 |
| 157 | Yemen | 57075.79 | 2203.97 | 1455.03 | 3340.95 | 3.44 | 2.27 | 5.22 |
| 160 | Afghanistan | 68619.81 | 29003.07 | 22249.91 | 43201.28 | 43.32 | 33.24 | 64.53 |
| 161 | Bangladesh | 108644.08 | 114390.70 | 91270.94 | 142202.70 | 9.34 | 7.45 | 11.61 |
| 162 | Bhutan | 147025.90 | 531.52 | 288.84 | 969.74 | 5.22 | 2.84 | 9.52 |
| 163 | India | 119801.09 | 1803940.94 | 1570354.17 | 2184333.34 | 15.84 | 13.79 | 19.18 |
| 164 | Nepal | 87724.85 | 20921.48 | 14980.91 | 27836.49 | 14.78 | 10.59 | 19.67 |
| 165 | Pakistan | 100826.18 | 296858.60 | 222148.79 | 365216.49 | 23.48 | 17.57 | 28.89 |
| 168 | Angola | 127535.04 | 78622.42 | 55270.44 | 103819.66 | 32.45 | 22.81 | 42.85 |
| 169 | Central African Republic | 53747.69 | 20614.40 | 13782.51 | 27860.64 | 333.07 | 222.69 | 450.15 |
| 170 | Congo | 114630.13 | 9708.35 | 6403.82 | 14279.21 | 28.75 | 18.96 | 42.29 |
| 171 | Democratic Republic of the Congo | 48912.54 | 110619.90 | 77609.21 | 176419.90 | 93.55 | 65.63 | 149.19 |
| 172 | Equatorial Guinea | 198829.70 | 1937.07 | 1170.49 | 3217.70 | 7.61 | 4.60 | 12.65 |
| 173 | Gabon | 202338.18 | 3655.80 | 2198.70 | 5416.88 | 10.87 | 6.54 | 16.10 |
| 175 | Burundi | 37422.47 | 11493.31 | 8792.27 | 14764.04 | 103.91 | 79.49 | 133.48 |
| 176 | Comoros | 76200.39 | 897.82 | 648.07 | 1209.64 | 35.05 | 25.30 | 47.22 |
| 177 | Djibouti | 105650.67 | 1786.72 | 1049.14 | 2646.76 | 23.87 | 14.01 | 35.35 |
| 178 | Eritrea | 59492.14 | 12660.92 | 8279.50 | 20175.48 | 100.47 | 65.70 | 160.10 |
| 179 | Ethiopia | 67339.20 | 84923.92 | 73300.54 | 99924.49 | 30.12 | 26.00 | 35.44 |
| 180 | Kenya | 100897.35 | 92491.62 | 56667.68 | 121989.08 | 34.61 | 21.20 | 45.64 |
| 181 | Madagascar | 53309.26 | 26597.06 | 19486.16 | 36153.96 | 58.23 | 42.66 | 79.15 |
| 182 | Malawi | 57996.89 | 17466.00 | 11852.53 | 25291.28 | 53.22 | 36.11 | 77.06 |
| 183 | Mauritius | 197105.53 | 123.29 | 109.08 | 137.10 | 0.43 | 0.38 | 0.47 |
| 184 | Mozambique | 56732.08 | 47384.20 | 33873.21 | 62562.07 | 104.65 | 74.81 | 138.17 |
| 185 | Rwanda | 68795.48 | 10252.45 | 6853.28 | 13800.31 | 28.29 | 18.91 | 38.08 |
| 186 | Seychelles | 227227.56 | 34.09 | 28.86 | 40.29 | 1.08 | 0.91 | 1.27 |
| 187 | Somalia | 57021.55 | 53467.46 | 32292.96 | 93652.02 | 177.71 | 107.33 | 311.28 |
| 189 | United Republic of Tanzania | 80618.64 | 52971.83 | 37853.71 | 70841.86 | 25.95 | 18.54 | 34.70 |
| 190 | Uganda | 72425.10 | 39990.88 | 29975.02 | 54933.40 | 34.39 | 25.77 | 47.24 |
| 191 | Zambia | 87867.16 | 13227.70 | 8840.58 | 20166.07 | 19.35 | 12.93 | 29.50 |
| 193 | Botswana | 211861.99 | 7641.16 | 5135.46 | 10467.05 | 17.78 | 11.95 | 24.35 |
| 194 | Lesotho | 88937.64 | 10427.02 | 7037.33 | 13324.37 | 218.60 | 147.54 | 279.34 |
| 195 | Namibia | 148998.91 | 6506.76 | 4431.38 | 9426.12 | 27.57 | 18.77 | 39.93 |
| 196 | South Africa | 181370.14 | 186769.61 | 167322.86 | 212968.17 | 23.96 | 21.46 | 27.32 |
| 197 | Kingdom of Eswatini | 172270.28 | 5411.30 | 3529.72 | 7774.64 | 47.57 | 31.03 | 68.35 |
| 198 | Zimbabwe | 87527.00 | 44582.99 | 29097.43 | 59734.98 | 89.76 | 58.58 | 120.27 |
| 200 | Benin | 81046.40 | 6731.81 | 4730.83 | 10487.48 | 14.39 | 10.12 | 22.43 |
| 201 | Burkina Faso | 70337.92 | 18704.46 | 13645.74 | 25269.37 | 33.05 | 24.11 | 44.65 |
| 202 | Cameroon | 99723.92 | 22883.56 | 14138.27 | 35673.87 | 15.02 | 9.28 | 23.42 |
| 203 | Republic of Cabo Verde | 108263.02 | 208.73 | 144.50 | 354.55 | 4.86 | 3.36 | 8.25 |
| 204 | Chad | 59130.01 | 19279.75 | 13974.13 | 25809.99 | 63.89 | 46.31 | 85.53 |
| 205 | Republic of C涔坱e d'Ivoire | 110227.82 | 24652.95 | 17255.77 | 36096.12 | 14.64 | 10.25 | 21.43 |
| 206 | Republic of the Gambia | 71577.29 | 1748.01 | 1279.22 | 2583.66 | 26.28 | 19.23 | 38.85 |
| 207 | Ghana | 112111.19 | 35907.70 | 26603.72 | 48699.98 | 15.87 | 11.76 | 21.52 |
| 208 | Guinea | 88240.20 | 11947.60 | 8451.45 | 17475.82 | 23.79 | 16.83 | 34.80 |
| 209 | Guinea-Bissau | 72040.01 | 2621.41 | 1924.70 | 3416.81 | 51.82 | 38.05 | 67.54 |
| 210 | Liberia | 53193.96 | 1954.79 | 1309.57 | 3597.07 | 23.26 | 15.58 | 42.81 |
| 211 | Mali | 69667.76 | 14003.36 | 10329.95 | 19945.30 | 24.75 | 18.25 | 35.25 |
| 212 | Mauritania | 97676.82 | 1673.06 | 1205.22 | 2599.19 | 6.52 | 4.70 | 10.13 |
| 213 | Niger | 53406.10 | 17998.17 | 12016.87 | 29918.60 | 45.33 | 30.27 | 75.36 |
| 214 | Nigeria | 105089.40 | 246036.18 | 165987.24 | 327089.10 | 19.38 | 13.08 | 25.77 |
| 215 | Sao Tome and Principe | 95100.92 | 67.01 | 47.66 | 108.90 | 5.40 | 3.84 | 8.77 |
| 216 | Senegal | 85843.66 | 9740.29 | 7304.55 | 13032.31 | 14.72 | 11.04 | 19.69 |
| 217 | Sierra Leone | 75600.36 | 8846.31 | 6441.96 | 13189.03 | 35.02 | 25.50 | 52.21 |
| 218 | Togo | 68517.76 | 5987.31 | 4261.69 | 9180.76 | 27.75 | 19.75 | 42.56 |
| 298 | American Samoa | 106290.41 | 5.02 | 4.20 | 6.03 | 1.44 | 1.21 | 1.73 |
| 305 | Bermuda | 392304.14 | 1.72 | 1.18 | 2.31 | 0.03 | 0.02 | 0.04 |
| 349 | Greenland | 361524.65 | 23.85 | 20.15 | 28.60 | 0.62 | 0.53 | 0.75 |
| 351 | Guam | 246206.35 | 80.60 | 70.01 | 92.82 | 1.33 | 1.16 | 1.53 |
| 367 | Principality of Monaco | 630546.54 | 6.98 | 5.44 | 8.60 | 0.09 | 0.07 | 0.11 |
| 369 | Republic of Nauru | 161601.18 | 15.78 | 10.94 | 21.38 | 11.74 | 8.13 | 15.90 |
| 376 | Northern Mariana Islands | 188960.57 | 29.19 | 24.46 | 35.60 | 2.87 | 2.41 | 3.51 |
| 380 | Republic of Palau | 169796.59 | 9.96 | 8.07 | 12.26 | 3.51 | 2.85 | 4.32 |
| 385 | Puerto Rico | 249005.44 | 120.85 | 101.96 | 140.74 | 0.09 | 0.08 | 0.11 |
| 393 | Saint Kitts and Nevis | 220129.71 | 6.26 | 5.26 | 7.46 | 0.40 | 0.33 | 0.47 |
| 396 | Republic of San Marino | 303761.15 | 0.74 | 0.50 | 0.99 | 0.03 | 0.02 | 0.05 |
| 416 | Tuvalu | 94141.44 | 14.63 | 10.58 | 18.29 | 22.10 | 15.98 | 27.63 |
| 422 | United States Virgin Islands | 283222.40 | 6.62 | 4.97 | 8.70 | 0.16 | 0.12 | 0.22 |
| 435 | South Sudan | 39199.69 | 11040.36 | 7739.27 | 16294.37 | 159.42 | 111.75 | 235.28 |
| 522 | Sudan | 72878.35 | 4255.57 | 2675.48 | 6263.26 | 3.02 | 1.90 | 4.44 |

Table 4. VLW and VLW/GDP by GBD regions in 2021 for TB, Generated Using Income Elasticity (IE) of the VSL at 0.55.

| Regions1 | Region | VLW region (millions) | VLW region (millions)  95%UI lower | VLW region (millions)  95%UI upper | VLW/GDP(%) | VLW/GDP(%) 95%UI lower | VLW/GDP(%) 95%UI upper |
| --- | --- | --- | --- | --- | --- | --- | --- |
| 1.00 | Global | 5164459.06 | 4163653.82 | 6520306.24 | 3.36 | 2.71 | 4.25 |
| 5.00 | High SDI | 70824.51 | 59605.31 | 85323.60 | 0.12 | 0.10 | 0.14 |
| 5.00 | High-middle SDI | 378224.55 | 316587.21 | 458374.28 | 0.75 | 0.62 | 0.90 |
| 5.00 | Low SDI | 715175.65 | 517452.33 | 1031429.87 | 37.74 | 27.31 | 54.43 |
| 5.00 | Low-middle SDI | 2937564.97 | 2375468.99 | 3674965.47 | 14.95 | 12.09 | 18.70 |
| 5.00 | Middle SDI | 1062669.39 | 894539.99 | 1270213.02 | 5.12 | 4.31 | 6.12 |
| 7.00 | Central Europe, Eastern Europe, and Central Asia | 135740.06 | 117191.41 | 157000.59 | 0.89 | 0.77 | 1.03 |
| 7.00 | High-income | 51542.14 | 45228.31 | 57353.33 | 0.09 | 0.08 | 0.10 |
| 7.00 | Latin America and Caribbean | 124155.25 | 105107.53 | 156753.65 | 1.06 | 0.89 | 1.33 |
| 7.00 | North Africa and Middle East | 62829.32 | 46067.74 | 93523.81 | 0.69 | 0.50 | 1.02 |
| 7.00 | South Asia | 2270943.73 | 1925151.77 | 2771028.48 | 15.74 | 13.35 | 19.21 |
| 7.00 | Southeast Asia, East Asia, and Oceania | 1104949.69 | 903222.82 | 1360826.95 | 2.80 | 2.29 | 3.45 |
| 7.00 | Sub-Saharan Africa | 1414298.87 | 1021684.26 | 1923819.43 | 29.26 | 21.14 | 39.80 |
| 21.00 | Andean Latin America | 31577.91 | 24237.44 | 40865.41 | 1.76 | 1.35 | 2.28 |
| 21.00 | Australasia | 560.24 | 498.32 | 619.01 | 0.03 | 0.03 | 0.04 |
| 21.00 | Caribbean | 12316.00 | 8261.16 | 27051.04 | 2.87 | 1.92 | 6.29 |
| 21.00 | Central Asia | 28212.33 | 23540.46 | 34130.08 | 1.97 | 1.64 | 2.38 |
| 21.00 | Central Europe | 13287.95 | 11358.93 | 15347.78 | 0.24 | 0.20 | 0.27 |
| 21.00 | Central Latin America | 29421.30 | 25268.75 | 34440.33 | 0.86 | 0.74 | 1.01 |
| 21.00 | Central Sub-Saharan Africa | 278428.63 | 192334.49 | 408901.11 | 42.82 | 29.58 | 62.88 |
| 21.00 | East Asia | 253608.65 | 204996.21 | 320910.98 | 0.87 | 0.70 | 1.10 |
| 21.00 | Eastern Europe | 94239.77 | 82292.02 | 107522.73 | 1.15 | 1.01 | 1.32 |
| 21.00 | Eastern Sub-Saharan Africa | 510352.25 | 359000.03 | 698022.83 | 41.04 | 28.87 | 56.13 |
| 21.00 | High-income | 32.55 | 26.76 | 39.51 | 0.18 | 0.15 | 0.22 |
| 21.00 | High-income Asia PaUIfic | 26550.82 | 22544.34 | 30177.23 | 0.30 | 0.25 | 0.34 |
| 21.00 | High-income North America | 9075.37 | 8388.58 | 9747.79 | 0.04 | 0.03 | 0.04 |
| 21.00 | Latin America and Caribbean | 127.10 | 107.22 | 148.20 | 0.09 | 0.08 | 0.11 |
| 21.00 | North Africa and Middle East | 62829.32 | 46067.74 | 93523.81 | 0.69 | 0.50 | 1.02 |
| 21.00 | Oceania | 11375.47 | 9117.63 | 14104.52 | 19.79 | 15.86 | 24.54 |
| 21.00 | South Asia | 2270943.73 | 1925151.77 | 2771028.48 | 15.74 | 13.35 | 19.21 |
| 21.00 | Southeast Asia | 839264.78 | 688492.99 | 1025024.37 | 9.79 | 8.03 | 11.96 |
| 21.00 | Southeast Asia, East Asia, and Oceania | 140.55 | 117.67 | 168.08 | 1.79 | 1.50 | 2.14 |
| 21.00 | Southern Latin America | 8525.79 | 7917.89 | 9182.32 | 0.46 | 0.43 | 0.50 |
| 21.00 | Southern Sub-Saharan Africa | 216755.84 | 187456.76 | 253960.35 | 25.14 | 21.74 | 29.45 |
| 21.00 | Tropical Latin America | 42187.16 | 39315.06 | 45066.34 | 1.02 | 0.95 | 1.09 |
| 21.00 | Western Europe | 15883.40 | 14268.62 | 17388.80 | 0.07 | 0.06 | 0.07 |
| 21.00 | Western Sub-Saharan Africa | 408762.15 | 282892.97 | 562935.14 | 19.68 | 13.62 | 27.10 |

Table 5. VLW and VLW/GDP by GBD regions in 2021 for TB, Generated Using Income Elasticity (IE) of the VSL at 1.50.

| Regions1 | Region | VLW region (millions) | VLW region (millions)  95%CI lower | VLW region (millions)  95%CI upper | VLW/GDP(%) | VLW/GDP(%) 95%CI lower | VLW/GDP(%) 95%CI upper |
| --- | --- | --- | --- | --- | --- | --- | --- |
| 1 | Global | 765423.2752 | 635669.616 | 937617.2925 | 0.498598641 | 0.414076782 | 0.610766255 |
| 5 | High SDI | 54796.52191 | 46174.36262 | 66126.53774 | 0.090587984 | 0.076334086 | 0.10931843 |
| 5 | High-middle SDI | 139511.5133 | 118598.0407 | 166173.519 | 0.275035808 | 0.233806567 | 0.327597823 |
| 5 | Low SDI | 27294.75611 | 19945.19288 | 38741.46193 | 1.440464661 | 1.052595794 | 2.044557811 |
| 5 | Low-middle SDI | 330122.6508 | 271113.6934 | 410652.3476 | 1.679969262 | 1.37967713 | 2.089778813 |
| 5 | Middle SDI | 213697.8331 | 179838.3265 | 255923.4262 | 1.029628423 | 0.866488208 | 1.233077705 |
| 7 | Central Europe, Eastern Europe, and Central Asia | 60630.64632 | 53515.46896 | 68529.56953 | 0.398305795 | 0.351563486 | 0.450196828 |
| 7 | High-income | 39477.75539 | 34818.89541 | 43827.07406 | 0.067349932 | 0.059401813 | 0.074769966 |
| 7 | Latin America and Caribbean | 31292.13535 | 27198.07043 | 36945.80412 | 0.266048138 | 0.231240083 | 0.314116064 |
| 7 | North Africa and Middle East | 22123.76989 | 16412.11863 | 32331.78516 | 0.241479892 | 0.179137491 | 0.352899892 |
| 7 | South Asia | 268088.9052 | 228781.8768 | 326260.1677 | 1.858709224 | 1.586186435 | 2.262021186 |
| 7 | Southeast Asia, East Asia, and Oceania | 227578.4846 | 186930.6261 | 279412.3255 | 0.576197818 | 0.47328296 | 0.707434064 |
| 7 | Sub-Saharan Africa | 116231.5784 | 88012.55971 | 150310.5663 | 2.404786998 | 1.82094627 | 3.109868251 |
| 21 | Andean Latin America | 6774.14958 | 5254.155845 | 8711.113894 | 0.378141447 | 0.293293508 | 0.486265201 |
| 21 | Australasia | 451.0106883 | 401.6445878 | 497.6260054 | 0.025795195 | 0.022971741 | 0.028461321 |
| 21 | Caribbean | 2240.198969 | 1609.587381 | 3850.90204 | 0.521171596 | 0.374462821 | 0.895893977 |
| 21 | Central Asia | 7211.023742 | 6111.383625 | 8615.365415 | 0.503276279 | 0.426529508 | 0.601288972 |
| 21 | Central Europe | 6970.22578 | 5990.493509 | 8011.479184 | 0.12397847 | 0.106552104 | 0.142499105 |
| 21 | Central Latin America | 7972.242491 | 6925.438399 | 9191.291129 | 0.233491581 | 0.202832712 | 0.269195161 |
| 21 | Central Sub-Saharan Africa | 16862.77788 | 11507.66646 | 23881.4603 | 2.593229374 | 1.769697667 | 3.672592071 |
| 21 | East Asia | 72808.19177 | 59219.12058 | 91445.80643 | 0.250097055 | 0.203418425 | 0.314117496 |
| 21 | Eastern Europe | 46449.3968 | 41413.59183 | 51902.72493 | 0.568731469 | 0.507072525 | 0.635502612 |
| 21 | Eastern Sub-Saharan Africa | 24224.33615 | 16739.39927 | 32566.81967 | 1.947857551 | 1.346000363 | 2.618669309 |
| 21 | High-income | 44.5719649 | 36.01361029 | 54.4016219 | 0.250620017 | 0.202497952 | 0.305890382 |
| 21 | High-income Asia Pacific | 18211.64667 | 15500.07148 | 20712.89475 | 0.204367508 | 0.173938746 | 0.232436021 |
| 21 | High-income North America | 8866.526686 | 8197.853613 | 9519.758437 | 0.034308687 | 0.031721282 | 0.036836342 |
| 21 | Latin America and Caribbean | 72.73156685 | 61.35627366 | 84.77052273 | 0.053797519 | 0.045383531 | 0.062702401 |
| 21 | North Africa and Middle East | 22123.76989 | 16412.11863 | 32331.78516 | 0.241479892 | 0.179137491 | 0.352899892 |
| 21 | Oceania | 754.3112341 | 603.7813317 | 936.3017115 | 1.31238189 | 1.050483738 | 1.629016451 |
| 21 | South Asia | 268088.9052 | 228781.8768 | 326260.1677 | 1.858709224 | 1.586186435 | 2.262021186 |
| 21 | Southeast Asia | 153505.9516 | 126655.762 | 186463.2025 | 1.791020419 | 1.477747628 | 2.175546939 |
| 21 | Southeast Asia, East Asia, and Oceania | 59.01933906 | 50.317593 | 69.38887048 | 0.752486167 | 0.641540439 | 0.884695864 |
| 21 | Southern Latin America | 3407.00758 | 3163.434149 | 3670.264446 | 0.184787066 | 0.171576289 | 0.199065421 |
| 21 | Southern Sub-Saharan Africa | 43037.33475 | 37592.33874 | 50147.89014 | 4.991094845 | 4.359631682 | 5.815715062 |
| 21 | Tropical Latin America | 10825.80516 | 10184.09839 | 11437.46209 | 0.26103526 | 0.245562222 | 0.275783727 |
| 21 | Western Europe | 12355.01007 | 11084.95671 | 13540.01926 | 0.051817133 | 0.046490506 | 0.056787083 |
| 21 | Western Sub-Saharan Africa | 32107.12963 | 22173.15524 | 43714.39618 | 1.545724186 | 1.067475752 | 2.104529437 |
